# The role of the immunoproteasome in interferon-γ-mediated microglial activation

**DOI:** 10.1038/s41598-017-09715-y

**Published:** 2017-08-24

**Authors:** Kasey E. Moritz, Nikki M. McCormack, Mahlet B. Abera, Coralie Viollet, Young J. Yauger, Gauthaman Sukumar, Clifton L. Dalgard, Barrington G. Burnett

**Affiliations:** 1 Neuroscience Program, Uniformed Services University of the Health Sciences, F. Edward Hébert School of Medicine, Bethesda, MD USA; 2 Department of Anatomy, Physiology and Genetics, Uniformed Services University of the Health Sciences, Bethesda, MD USA; 3 Collaborative Health Initiative Research Program, Uniformed Services University of the Health Sciences, Bethesda, MD USA; 4 The American Genome Center, Uniformed Services University of the Health Sciences, Bethesda, MD USA

## Abstract

Microglia regulate the brain microenvironment by sensing damage and neutralizing potentially harmful insults. Disruption of central nervous system (CNS) homeostasis results in transition of microglia to a reactive state characterized by morphological changes and production of cytokines to prevent further damage to CNS tissue. Immunoproteasome levels are elevated in activated microglia in models of stroke, infection and traumatic brain injury, though the exact role of the immunoproteasome in neuropathology remains poorly defined. Using gene expression analysis and native gel electrophoresis we characterize the expression and assembly of the immunoproteasome in microglia following interferon-gamma exposure. Transcriptome analysis suggests that the immunoproteasome regulates multiple features of microglial activation including nitric oxide production and phagocytosis. We show that inhibiting the immunoproteasome attenuates expression of pro-inflammatory cytokines and suppresses interferon-gamma-dependent priming of microglia. These results imply that targeting immunoproteasome function following CNS injury may attenuate select microglial activity to improve the pathophysiology of neurodegenerative conditions or the progress of inflammation-mediated secondary injury following neurotrauma.

## Introduction

Microglia are the primary inflammatory mediators of the central nervous system (CNS). Damage to the CNS results in the transition of microglia from a surveying or ‘ramified’ state, to a ‘reactive’ state, allowing them to respond to changes in the local milieu^1–3^. The early activation response of microglia is characterized by the production of pro-inflammatory cytokines and cytotoxic factors that exacerbate neuroinflammation and promote cell death, respectively^4^. Microglial activation occurs rapidly, over the course of hours, and is usually transient but can persist for days or years^5–7^. How this transition occurs is unknown but of tremendous interest in modulating the immune response.

One potential mediator of the immune response in microglia is the proteasome. The ubiquitin proteasome system (UPS) is one of the major regulators of cellular homeostasis. Under standard conditions, the constitutive (26S) proteasome is the primary regulator of intracellular protein degradation^8^. In response to inflammation or cellular stress, the UPS displays considerable plasticity that allows it to adapt rapidly and dynamically. An alternate form of the proteasome, the immunoproteasome (20i), is assembled, resulting in alterations to protein processing^9^. The immunoproteasome is distinguished from the constitutive proteasome by the presence of immuno-subunits β1i, β2i and β5i rather than constitutive catalytic subunits β1, β2 and β5, respectively^10^ and the 11 S regulatory lid components PA28α and β^11, 12^.

*De novo* immunoproteasome formation varies by cell type, but typically faster than formation of the constitutive proteasome^13^. In microglia, immunoproteasomes are detectable as early as 4 h following stroke and recent reports have shown that increased levels of immunoproteasomes in microglia correlate with increased levels of pro-inflammatory markers^14^. Furthermore, inhibiting the proteasome decreases activation of microglia in stroke, infection and traumatic brain injury (TBI), resulting in decreased lesion volume, reduced neuroinflammation, and improvements in behavioral deficits^14–17^. Despite the overwhelming evidence suggesting that altered proteasome dynamics impact the microglial response during inflammation, no studies to date have described the rapid immunoproteasome assembly mechanism nor how immunoproteasomes impact microglial function.

In the current study, we show that microglia stimulated with interferon gamma (ΙFNγ) transition to a reactive state accompanied by induction and assembly of the immunoproteasome. We provide evidence that pharmacological or genetic immunoproteasome inhibition is sufficient to suppress the response of microglia to a second stimuli, lipopolysaccharide (LPS). Immunoproteasomes have been linked to inflammatory diseases and cancers in humans, thus making them a potential therapeutic target for a range of pathological conditions. Data from this study indicate that targeting the immunoproteasome during neuroinflammation could attenuate select microglial activity to improve the pathophysiology of neurodegenerative conditions or the progress of inflammation-mediated secondary injury following neurotrauma.

## Results

### The immunoproteasome is up-regulated following TBI

**Figure 1:**
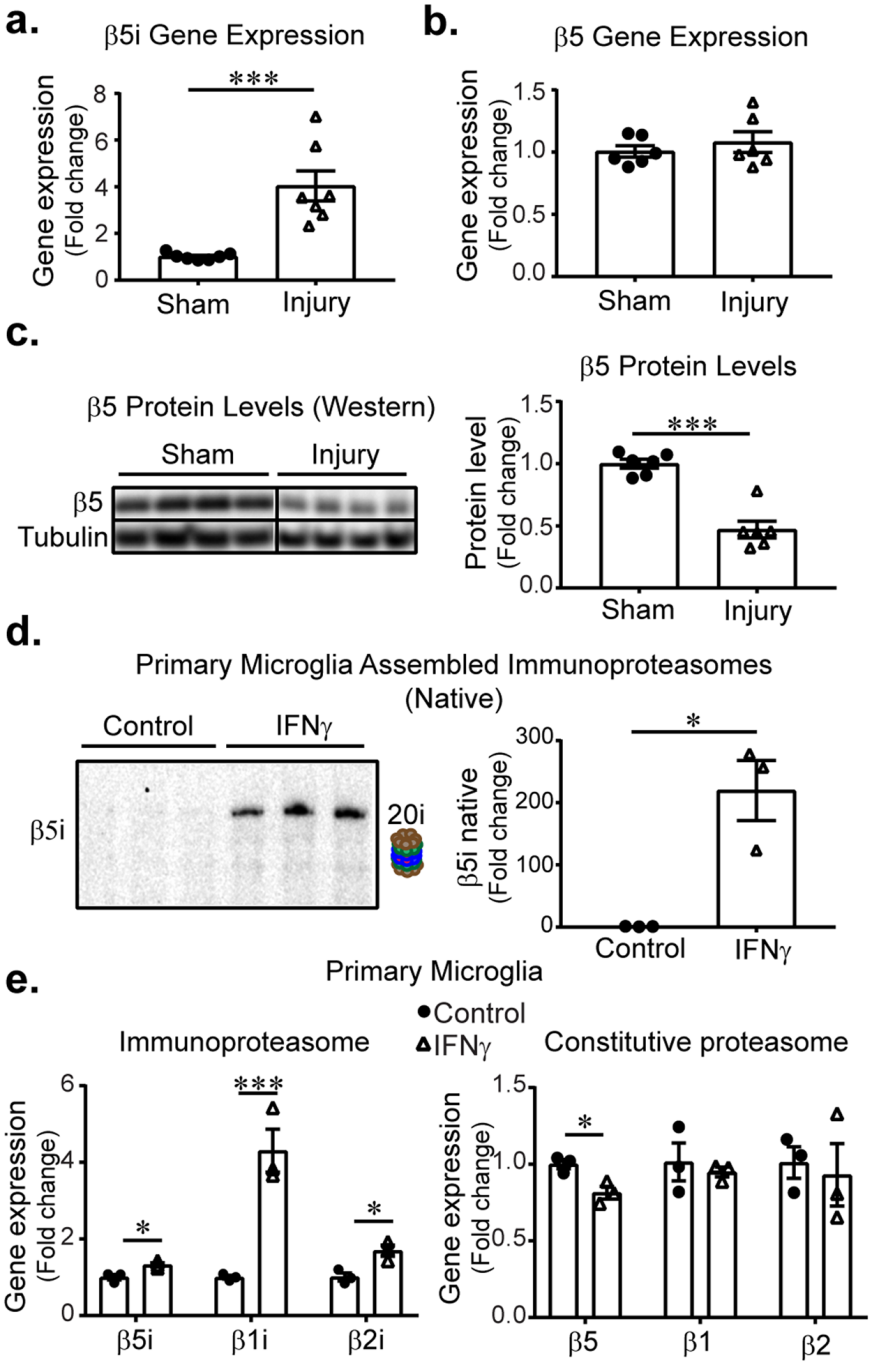
Altered proteasome dynamics in brains and microglia. (**a**) Animals subjected to CCI contain significantly higher gene expression of immunoproteasome catalytic subunit β5i compared to sham animals (t(12) = 4.69, *p* < 0.001, n = 7). (**b**) There is no difference in constitutive proteasome subunit β5 (t(10) = 0.7837, *p* = 0.451). (**c**) Injured animals had significantly reduced β5 protein levels (t(10) = 7.024, *p* < 0.001, n = 6/treatment group). (**d**) Primary microglia treated with IFNγ for 24 h (n = 3/group) have significantly higher assembled immunoproteasomes than controls (t(2.001) = −4.757, *p* = 0.041). (**e**) Gene expression of immunoproteasome subunits β5i (t(2.001) = −4.757, *p* = 0.024), β1i (t(2.030) = −5.856, *p* = 0.027), and β2i (t(4) = −3.879, *p* = 0.018) are all significantly increased. Constitutive proteasome subunit β5 is reduced (t(4) = 3.640, *p* = 0.022), whereas the others remain unchanged (β1:t(4) = 0.510, *p* = 0.637; β2:t(4) = 0.352, *p* = 0.743). Data are presented as relative fold change compared to sham animals or control cells, analysed by independent samples t-test.

Microglia, as the principle immune effectors in the brain, undergo a conversion into a reactive pro-inflammatory phenotype in neurotrauma rapidly^18–20^.The immunoproteasome is thought to be up-regulated following injury to protect tissues from damage by mediating the inflammatory response of immune cells. We first sought to determine if this was true in a mouse model of TBI. Using the controlled cortical impact (CCI) injury model we performed a gene profiling analysis to assess the expression of the constituent proteasome subunits from sham and CCI-injured mice 24 h following injury. We found higher expression of the immunoproteasome catalytic subunits Psmb8 (β5i), Psmb9 (β1i) and Psmb10 (β2i) at the injury site compared to the same region of their sham-injured counterparts (Fig. 1a and Supplemental Fig. S1). Gene expression of the constitutive catalytic subunit Psmb5 (β5) and core subunit Psma3 (α7) was unchanged while β5 protein was reduced (Fig. 1b,c and Supplemental Fig. S1), consistent with a previous study^21^. We also found that IFNγ, the primary mediator of immunoproteasome induction, was up-regulated following injury (Supplemental Fig. S1). Thus, the immunoproteasome is rapidly induced in the brain of mice after injury, presumably to protect the CNS from further damage as suggested in ischemic stroke and infections^14, 22^.

Given microglia are the resident immune cells of the brain, we next sought to determine if the rapid shift in proteasome subtypes occurs in microglia. We treated rat primary microglia (purity confirmation in Supplemental Fig. S2) with IFNγ for 24 h, then performed native gel electrophoresis to examine assembled immunoproteasome levels. We found that immunoproteasomes are assembled in microglia within 24 h of IFNγ exposure (Fig. 1d). Intriguingly, IFNγ induced expression of immunoproteasome catalytic subunits but did not alter global expression of other core proteasome subunits, in line with results from injured brain extracts (Fig. 1e).

**Figure 2:**
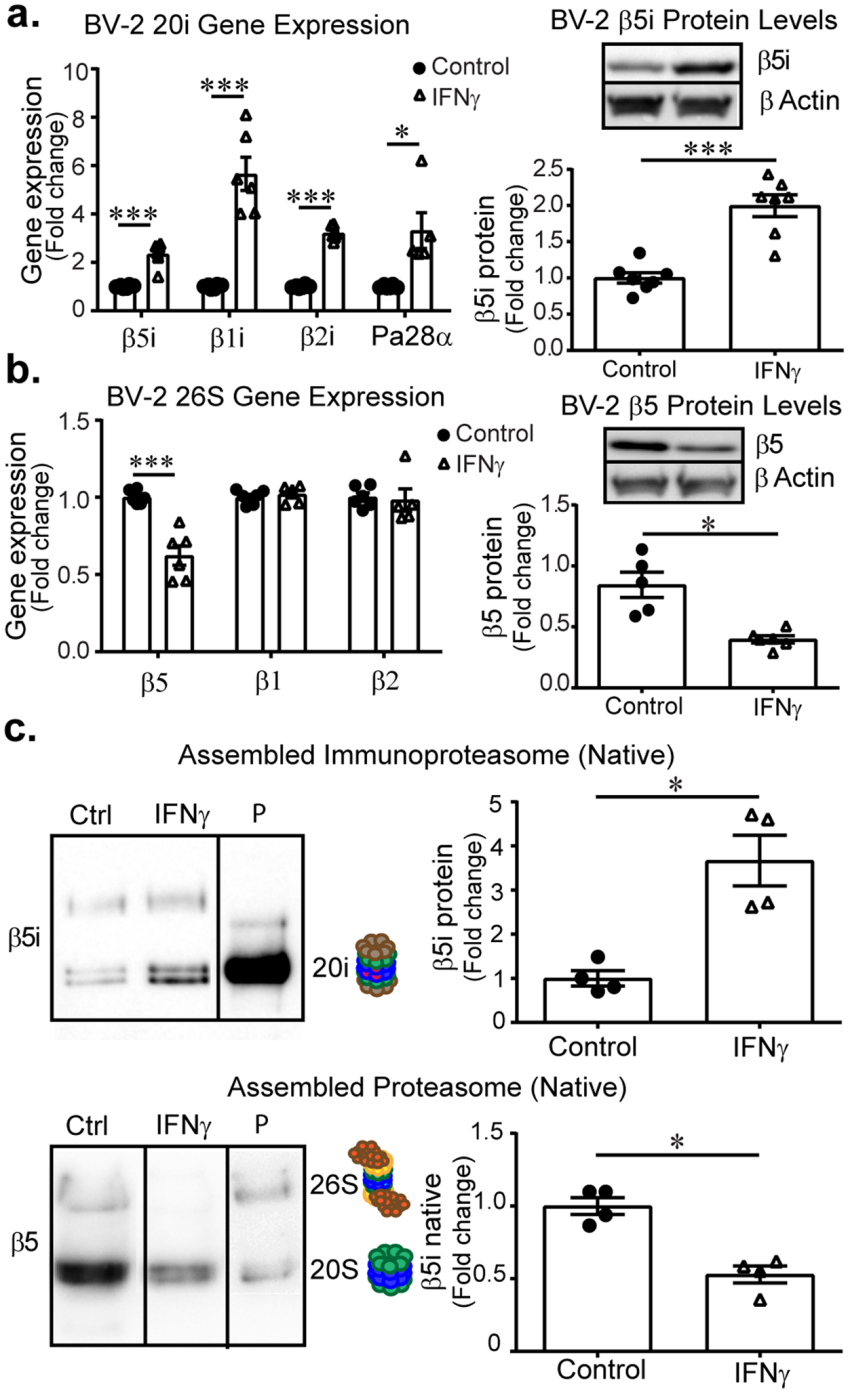
Immunoproteasomes in BV-2 microglia. BV-2 cells were subjected to 24 h IFNγ and prepared for gene expression (n = 6) and protein (n = 7) analysis. (**a**) Immunoproteasome genes are significantly increased following treatment (β5i: t(5.145) = −6.524, *p* = 0.001; β1i: t(5.024) = −6.795, *p* = 0.001; β2i: t(5.532) = −17.691, *p* < 0.001; pa28α: t(4.010) = −3.143, *p* = 0.035) accompanied by increased β5i protein levels (t(12) = −6.006, *p* < 0.001). (**b**) Gene expression analysis reveals that β5 (t(5.783) = 5.745, *p* = 0.001) is significantly decreased, however no other core subunits catalytic subunits are changed (β1:t(9) = −0.647, *p* = 0.534; β2:t(6.011) = −0.469, *p* = 0.656). Total protein levels of the β5 catalytic subunit is significantly reduced following treatment with IFNγ (t(4.637) = 4.129, *p* = 0.011). (**c**). Assembled immunoproteasomes are significantly increased (n = 4) following IFNγ treatment (t(3.543) = −4.452, *p* = 0.015). Assembled constitutive proteasomes are reduced (n = 4) after IFNγ treatment (t(6) = 5.675, *p* = 0.001). Data are presented as relative fold change compared to untreated control cells, analysed by independent samples t-test. (P-purified proteasome).

To further dissect proteasome molecular assembly dynamics we examined immunoproteasome assembly in BV-2 cells, an immortalized murine microglia cell line that has been shown to replicate the functional response to IFNγ treatment^23, 24^. We treated BV-2 cells with 200 U/mL IFNγ for 24 h and found up-regulation of immunoproteasome catalytic subunits β5i, β1i, β2i and the regulatory lid component PA28α, consistent with results obtained in primary microglia. Also, we confirmed β5i protein was increased by western blot analysis (Fig. 2a). Gene expression of constitutive catalytic subunits β5, β1, and β2, were slightly reduced or unchanged (Fig. 2b). While the induction of immunoproteasome subunits occurred within 24 h of IFNγ treatment, it was unclear if the composition of fully assembled proteasomes was also altered. To measure changes in assembled proteasomes, we performed native gel electrophoresis on BV-2 cell lysates collected 24 h after IFNγ treatment. The levels of assembled immunoproteasome increased while the levels of assembled constitutive proteasome decreased following IFNγ treatment (Fig. 2c). Given the proteasome subtype conversion we next determined whether proteasome catalytic activity was also altered in response to IFNγ. Proteasome catalytic activity from cells treated with IFNγ was measured using the Proteasome-Glo cell-based assay and confirmed in cell extracts using a fluorogenic substrate. We found proteasome activity was unchanged following IFNγ treatment (Supplemental Fig. S3) despite subtype conversion. Together these results demonstrate that immunoproteasomes can rapidly replace constitutive proteasomes in response to IFNγ with no apparent induction of core proteasome subunits.

### Pomp mediates the interferon-γ-dependent alteration in proteasome composition in BV-2 cells

**Figure 3:**
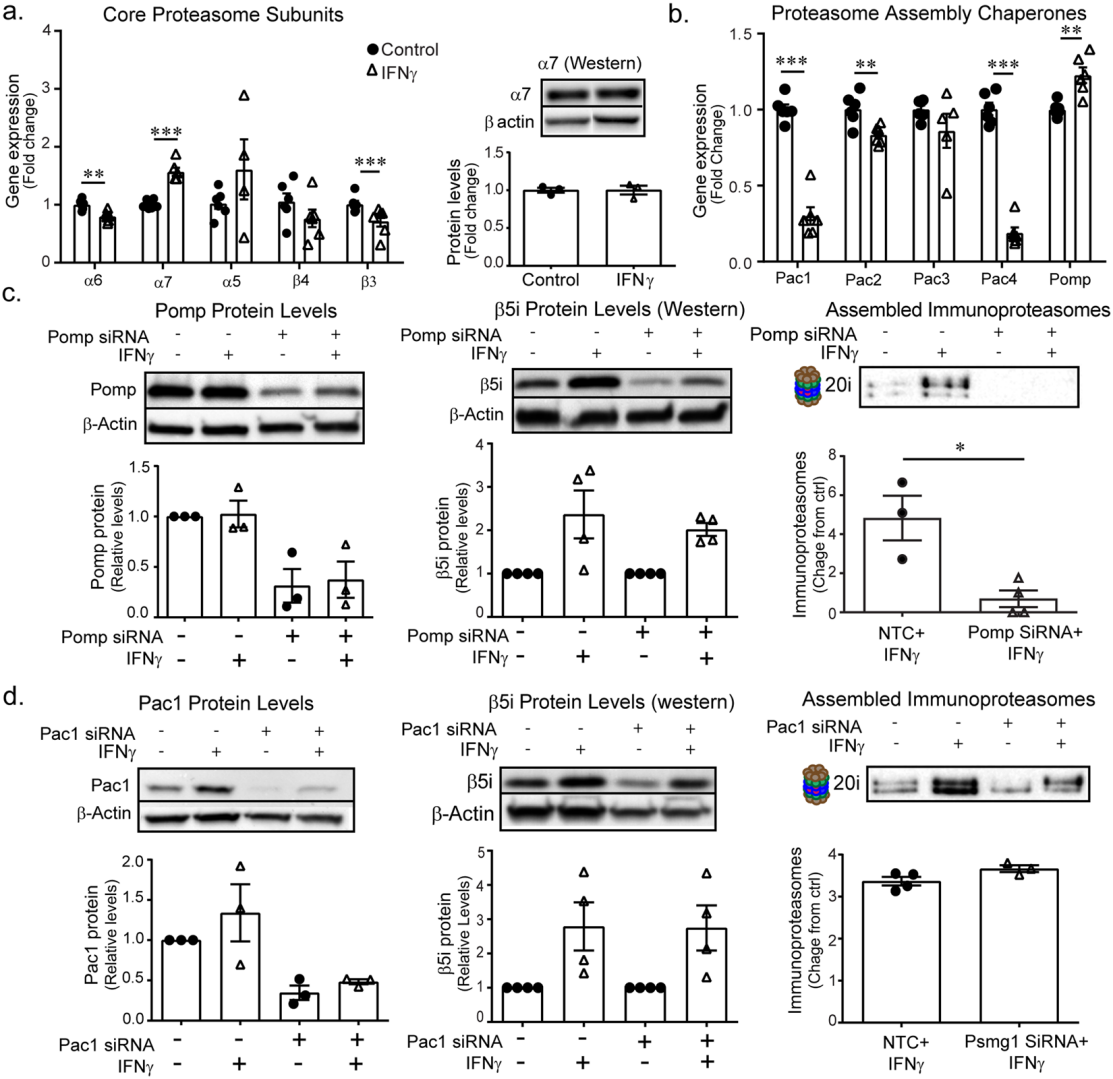
24 h immunoproteasome formation is Pomp-dependent. (**a**) BV-2 cells treated with IFNγ were examined for *de novo* immunoproteasome production (n = 6). Gene expression of non-catalytic core subunits is reduced (α6: t(10) = 4.013, *p* = 0.002; β3: t(10) = 2.583, *p* = 0.027) or unchanged (α5: t(3.197) = −1.112, *p* = 0.343; β4: t(6.011) = −0.469, *p* = 0.186) relative to controls. Despite a modest rise in α7 gene expression (t(10) = −7.561, *p* < 0.001), protein levels are unchanged. (**b**) Gene expression of assembly chaperones Pac1 (t(10) = 10.650, *p* < 0.001), Pac2 (t(10) = 3.548, *p* = 0.005) and Pac4 (t(10) = 14.869, *p* < 0.001) is significantly reduced following IFNγ treatment. Pac3 is unchanged (t(9) = 1.336, *p* = 0.214). Late assembly chaperone and proteasome maturation protein, Pomp was significantly increased following treatment (t(10) = −4.158, *p* = 0.002). (**c)**. We performed siRNA-mediated knockdown of Pomp and measured fold change of immunoproteasome induction in response to IFNγ. Pomp protein levels were significantly changed following knockdown (F(3,8) = 7.730, *p* = 0.009, one-way ANOVA, n = 3) and IFNγ-dependent immunoproteasome assembly is reduced (t(5) = 3.814, *p* = 0.012, t-test comparing IFNγ-induced fold-change of NTC (n = 3) vs. Pomp siRNA (n = 4)). (**d**). Pac1 siRNA-mediated gene silencing was sufficient to reduce Pac1 levels compared to NTC (F (3,8) = 6.198, *p* = 0.018, n = 3), however did not impact immunoproteasome assembly in response to IFNγ (t(3.076) = −1.318, *p* = 0.277, t-test comparing IFNγ-induced fold change of NTC vs. IFNγ-induced fold change of Pac1 siRNA knockdown, n = 4).

The proteasome displays considerable plasticity that allows it to adapt rapidly to multiple cellular challenges. The immunoproteasome is normally poorly expressed in the CNS^25^, but can be induced under pathological conditions^26^. However, what determines the relative levels of each type of proteasome and the physiological relevance of the redistribution has not been established. Thus, we sought to determine the mechanism through which IFNγ alters proteasome composition in microglia. *De novo* immunoproteasome assembly requires induction and nascent synthesis of α_1−7_ and β_1−7_ subunits. We found that unlike the catalytic subunits, the core non-catalytic α and β proteasome subunits were not altered in microglia in response to IFNγ, at the time when we detect assembled immunoproteasomes by native-gel electrophoresis (Fig. 3a), consistent to our findings in the brain of mice 24 h after TBI. Moreover, western blot analysis confirmed that core subunit protein (α7) level is unchanged at this time point (Fig. 3a). Without induction of the core subunits, we thought it is more likely that immunoproteasome catalytic subunits replaced the constitutive catalytic subunits in pre-existing constitutive proteasomes.

To test this hypothesis, we examined gene expression of known proteasome assembly chaperones. *De novo* immunoproteasome assembly begins with the formation of the α ring, facilitated by Pac1/Pac2 (Psmg1/Psmg2) heterodimers^27, 28^, followed by Pac3/Pac4 (Psmg3/Psmg4)-mediated β ring assembly^29^. Interestingly, we found that, although assembled immunoproteasomes are present in BV-2 cells exposed to IFNγ, these cells exhibit reduced gene expression of the chaperones (Pac1, 2 and 4) that mediate *de novo* proteasome assembly but a slight increase in the proteasome maturation factor Pomp (Fig. 3b). Given the role of Pomp in late stage proteasome formation and maturation^13, 30^, we performed siRNA-mediated Pomp knockdown and measured immunoproteasome assembly following IFNγ treatment. We found that Pomp knockdown nearly abolished IFNγ-induced immunoproteasome assembly (Fig. 3c). Since Pac1 is required for *de novo* immunoproteasome assembly^27^, we performed siRNA-mediated knockdown of Pac1 to determine if it was necessary for IFNγ-induced immunoproteasome formation at 24 h. While Pac1 knockdown reduced overall proteasome levels compared to scrambled controls, the IFNγ-induced increase in assembled immunoproteasomes was not changed (Fig. 3d). Together, these data are consistent with Pomp-dependent immunoproteasome assembly in microglia following IFNγ treatment.

### Immunoproteasome modulation via Janus kinase (JAK)

**Figure 4:**
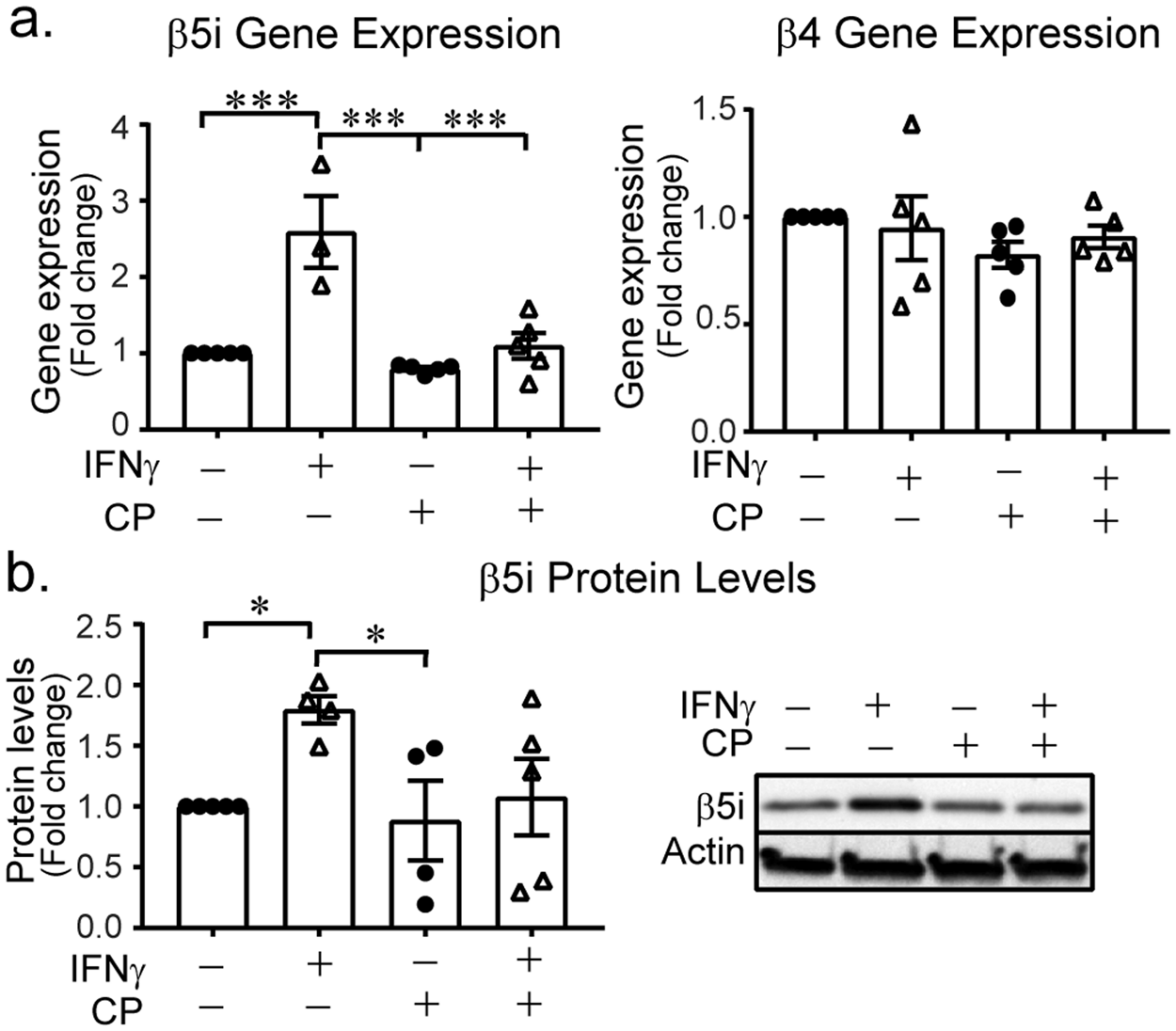
Immunoproteasome induction is mediated by Jak3 inhibition. BV-2 cells were treated with IFNγ in the absence and presence of Jak3 inhibitor, CP-690550 (CP, 10 µM) for 24 h. (**a**) Gene expression analysis revealed a significant effect of drug treatment on β5i gene expression (F(3,15) = 5.931, *p* = 0.007, n = 5). IFNγ-induced (n = 3) β5i expression was higher than control (n = 5, *p* = 0.016), CP-690550 (n = 5, *p* = 0.010) and IFNγ + CP-690550 (n = 5, *p* = 0.020). CP-690550 co-treatment with IFNγ does not increase β5i levels (*p* = 0.999, compared to control). Jak3 inhibition not have a global effect on all proteasome subunits, as gene expression analysis revealed no change in a non-catalytic core subunit, β4 (F(3,15) = 1.834, *p* = 0.184, n = 5). (**b**) β5i protein levels were significantly different between groups (F(3,14) = 4.316, *p* = 0.024). IFNγ (n = 4) significantly increases protein levels of β5i protein (*p* = 0.041) compared to control (n = 5). IFNγ does not increase immunoproteasome protein levels in cells that are simultaneously treated with CP-690550 (*p* = 0.998, compared to control, n = 5). Data are presented as relative fold-change compared to untreated control cells, analysed by one-way ANOVA with Tukey’s *post hoc* test.

IFNγ signals through the JAK/STAT signal transduction pathway and JAK is a major inflammatory transcription factor in hematopoietic cells. We thus sought to determine if we could block the IFNγ−dependent induction of the immunoproteasome by inhibiting JAK signaling. CP-690550 is a JAK3 inhibitor that is currently in clinical trials for treatment of rheumatoid arthritis, juvenile idiopathic arthritis, ulcerative colitis and other inflammatory conditions. We found that CP-690550 treatment blocked gene induction and protein expression of the immunoproteasome subunits in cells co-treated with IFNγ (Fig. 4a and b). To confirm that CP-690550 did not globally reduce all proteasome subunits, we performed gene expression analysis on the β4 core subunit. CP-690550 did not alter subunit β4 gene expression (Fig. 4a). Moreover, immunoproteasome induction was also suppressed by the JAK2 inhibitor AZD1480 (Supplemental Fig. S4), further validating the JAK signaling pathway in mediating proteasome dynamics in microglia. These data suggest that immunoproteasome assembly is mediated by JAK signaling and could provide mechanistic insight into the pathways targetd by JAK inhibitors in the treatment of inflammation.

### Interferon-γ transcriptome changes are reversed by immunoproteasome inhibition

**Figure 5:**
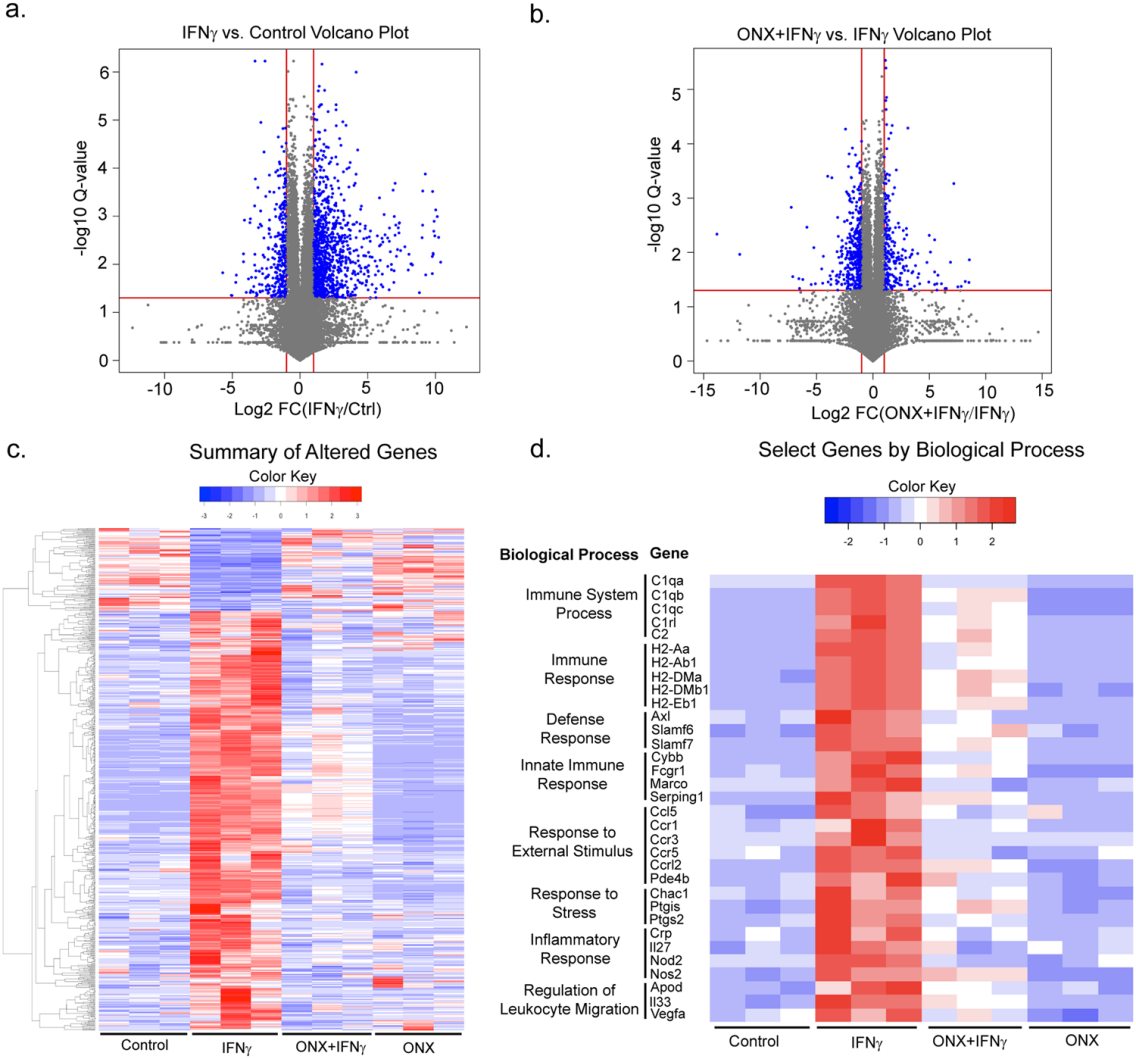
Transcriptome changes in BV-2 cells. BV-2 cells were treated with IFNγ and/or ONX-0914 for 24 h and transcriptome profiling by RNA-seq was performed. (**a**) Differential expression of 3,482 genes after exposure to IFNγ. (**b**) Differential expression of 2,670 genes between IFNγ exposed and IFNγ and ONX-0914 exposed cells. (**c**) Expression signature of 703 differential expressed genes by IFNγ exposure that are reversed with co-administration of ONX-0914. (**d**) Expression signature of immune response enriched genes with differential expression after IFNγ exposure and reversal by co-administration of ONX-0914. Gene ontologies associated with biological processes are enriched by overlapping gene sets and fold-enrichment (Table 2).

To better understand the functional consequences of immunoproteasome induction on microglia activity, we sought to identify IFNγ-associated transcriptome changes that are reversed in the presence of the immunoproteasome inhibitor ONX-0914. In IFNγ-exposed microglia as compared to vehicle-treated controls, we observed a significantly differential signature of 3,482 genes (2,677 up-regulated and 805 down-regulated genes; Fig. 5a, Table 1). The IFNγ-associated transcriptome signature was enriched for canonical cellular activities involved in the immune response and inflammation^23, 31^. We also observed 2,670 genes that were differentially expressed in microglia with IFNγ and ONX-0914 co-exposure, as compared to IFNγ exposure alone (1,381 up-regulated and 1,289 down-regulated genes, Fig. 5b, Table 1). In confirmation of our *q*PCR results, the expression of all three immunoproteasome catalytic subunits were increased after exposure with IFNγ and no change in the expression of the non-catalytic subunits was observed (Supplemental Table S2).

**Table 1:** List of genes significantly altered (up or down) by IFNγ and by ONX-0914 + IFNγ.

	IFNγ vs Control	ONX-0914 + IFNγ vs IFNγ
Total # genes altered	3,482	2,670
# Gene up-regulated	2,677	1,381
# Gene down-regulated	805	1,289

Additionally, we found 703 genes that were altered by IFNγ and subsequently abrogated when the immunoproteasome inhibitor (ONX-0914) was co-administered (Supplemental Table S3). 588 of these were up-regulated with IFNγ and down-regulated with ONX-0914, while 115 were down-regulated with IFNγ and up-regulated with ONX-0914 (Fig. 5c). Functional enrichment analysis for these 588 genes revealed that immunoproteasome inhibition suppressed cellular activation for inflammatory processes (99 genes total in 6 ontological processes, Fig. 5d, Supplemental Table S4, Table 2). Prominent among the genes that were up-regulated by IFNγ but reduced with ONX-0914 were the key inflammatory mediators Nos2 (iNos), Cybb (Nox2) and Ptgs2 (Cox-2). The major histocompatibility complex (MHC), critical for adaptive immune responses, is also suppressed by ONX-0914 in IFNγ-stimulated microglia and functionally significant, given the role of the proteasome in antigen presentation.

**Table 2:** Biological processes enriched for genes up-regulated with IFNγ and down-regulated with ONX-0914.

Enriched Biological Processes	# genes	Fold Enrichment	P-value
Immune System Process	91	2.45	1.23E-11
Immune Response	64	3.05	2.91E-11
Defense Response	65	2.95	8.46E-11
Innate Immune Response	45	3.85	2.32E-10
Inflammatory Response	26	3.26	1.82E-03
Regulation of Leukocyte Migration	16	4.94	2.26E-03

Furthermore, we also observed a subset of genes whose expression was not reversed by immunoproteasome inhibition (Supplemental Table S5). Of these 689 genes, 41 were involved in inflammatory response including 12 anti-inflammatory genes such as Serpinf1, Arg1, Cd163, and Il20rb. Also in this group were genes shown to be involved in phagocytosis including CD68, Spp1, Fcgr2b, and Mrc1which were down-regulated by IFNγ. Our findings would suggest that many of the IFNγ−dependent genes for inflammatory activation require the immunoproteasome. In contrast the anti-inflammatory pathways or pathways involved in phagocytosis were either exacerbated or unaffected by immunoproteasome inhibition. Together, the above results identify the immunoproteasome as a regulator of multiple features of the microglial pro-inflammatory profile.

### Loss of immunoproteasomes decreases microglia inflammatory response

**Figure 6:**
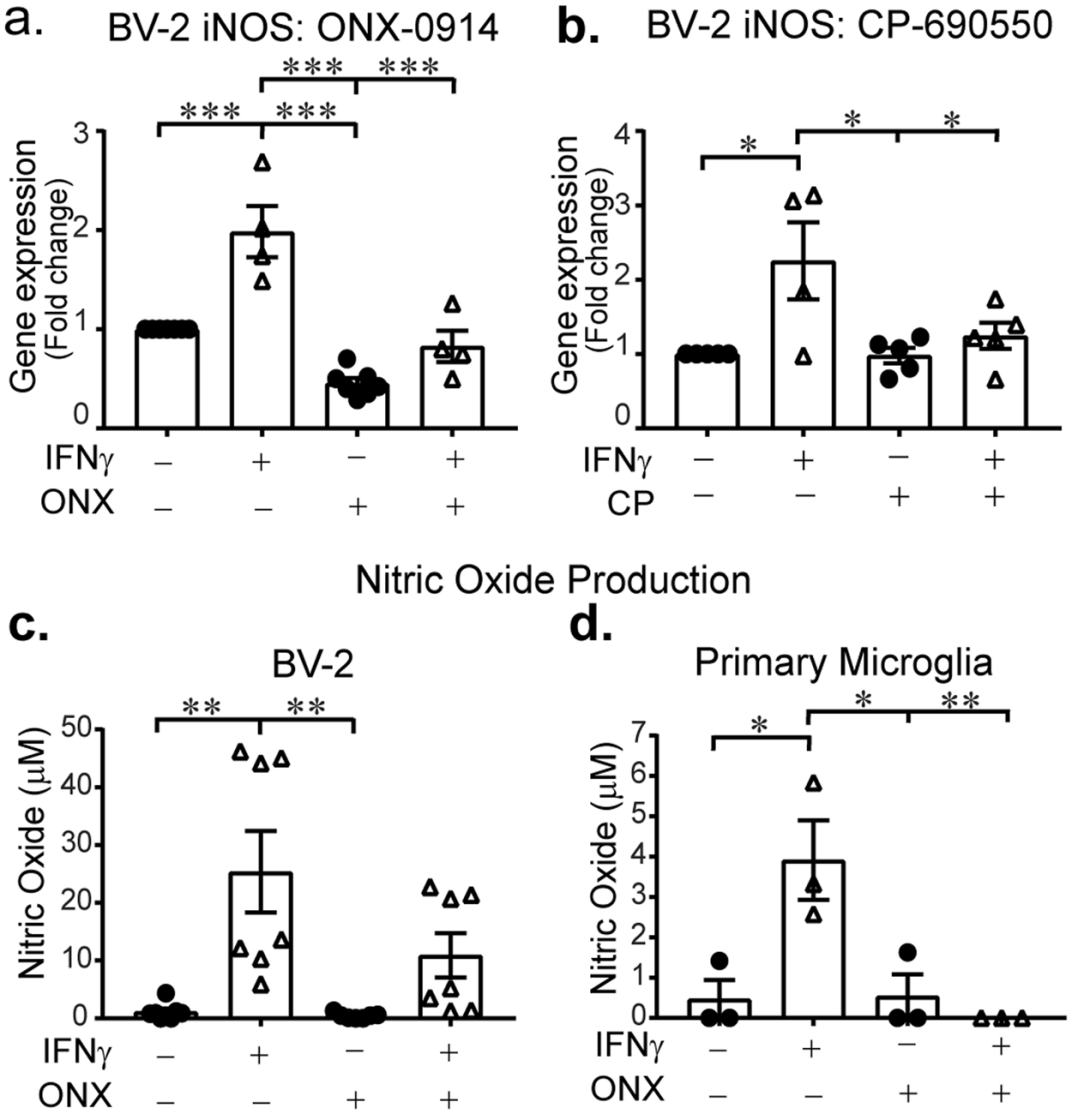
Immunoproteasomes modulate IFNγ-induced inflammation response. BV-2 cells and primary microglia were treated with IFNγ in the absence and presence of immunoproteasome (ONX-0914) and JAK3 (CP-690550, CP) inhibitors, then iNOS and NO levels were measured. (**a**) Treatment significantly alters iNOS gene expression (F(3,19) = 31.437, *p* < 0.001). IFNγ (n = 4) significantly increases iNOS gene expression compared to control (n = 5, *p* < 0.001), whereas IFNγ co-treatment with ONX-0914 (n = 4) results in reduced levels of iNOS gene expression compared to IFNγ alone (*p* < 0.001). Bars represent mean fold change compared to normalized control. (**b**) IFNγ and CP-690550 treatment significantly impacts iNOS gene expression (F(3,14) = 16.353, p < 0.001). IFNγ (n = 4) significantly increases iNOS compared to control (n = 5, *p* < 0.001). IFNγ alone results in significantly higher iNOS gene expression than CP-690550 treatment (n = 5, *p* = 0.049) and IFNγ + CP-690550 co-treatment (n = 5, *p* = 0.001). Bars represent mean fold change compared to normalized control. (**c**) ONX-0914 treatment has a significant effect on NO levels in BV-2 cells (F(3,24) = 68.354, *p* < 0.001, n = 7). IFNγ treatment increases levels of NO compared to control (*p* = 0.001). Co-treatment with IFNγ and ONX-0914 results in significantly lower NO production compared to IFNγ alone (*p* = 0.001). (**d**) NO levels in primary microglia are significantly different between groups (F(3,8) = 8.717, *p* = 0.006, n = 3). IFNγ significantly increased NO levels compared to control (*p* = 0.017), ONX-0914 (*p* = 0.019) and co-treatment (*p* = 0.008).

We next sought to functionally validate results from our transcriptome analysis. The enzyme nitric oxide synthase (NOS) was of interest because of its key role in mediating neuroinflammation^32, 33^. To determine if immunoproteasome inhibition could decrease microglia-mediated inflammation we examined Nos2 (iNOS) gene expression and nitric oxide (NO) levels 24 h following IFNγ treatment. Consistent with our transcriptome data we found iNos is up-regulated in microglia exposed to IFNγ (Fig. 6a). Treatment of BV-2 cells with immunoproteasome inhibitor ONX-0914 resulted in significantly reduced iNos gene expression (Fig. 6a). Further, since CP-690550 reduced immunoproteasome levels, we measured iNos gene expression in BV-2 cells in the absence and presence of the JAK inhibitor and found that iNOS and NO levels were reduced compared to IFNγ alone (Fig. 6b and Supplemental Fig. S4). Elevated production of the iNos gene product NO can result in cell toxicity and has been implicated in neurodegenerative conditions, emphasizing the importance of decreasing NO production in treating brain injury^34^. We found that ONX-0914 significantly reduced IFNγ-dependent NO production in both BV-2 cells and primary microglia (Fig. 6c and d, respectively). To ensure that the effect of ONX-0914 on NO production were not due to off-target effects, we created a Psmb8 (β5i) Crispr/Cas9n knock-out BV−2 cell line (BV-2 β5i KO). These cells lack the catalytic β5i proteasome subunit (Supplemental Fig. S5), the pharmacological target of ONX-0914. We found that BV-2 β5i KO cells produced lower levels of NO in response to IFNγ (Supplemental Fig. S5b). To rule out the possibility that the β5i BV2 KO cell line lost the ability to respond to IFNγ we next examined CD40, a co-stimulatory protein whose primary activation signal is IFNγ. Flow cytometry analysis revealed that both normal BV-2 and β5i BV-2 KO cells displayed similar increased mean fluorescence intensity (MFI) following IFNγ treatment compared to untreated cells (Supplemental Fig. S5). These data are consistent with our transcriptome analysis showing IFNγ−dependent CD40 gene induction is unaffected by ONX-0914. Immunoproteasome inhibition has been shown to reduce inflammation in rodent models of rheumatoid arthritis, muscular dystrophy, multiple sclerosis and stroke^14, 35–37^. We show here that blocking immunoproteasome assembly or inhibiting its activity reduces IFNγ-dependent NO release, highlighting one potential inflammatory mechanism.

### Immunoproteasome inhibition decreases phagocytosis

**Figure 7:**
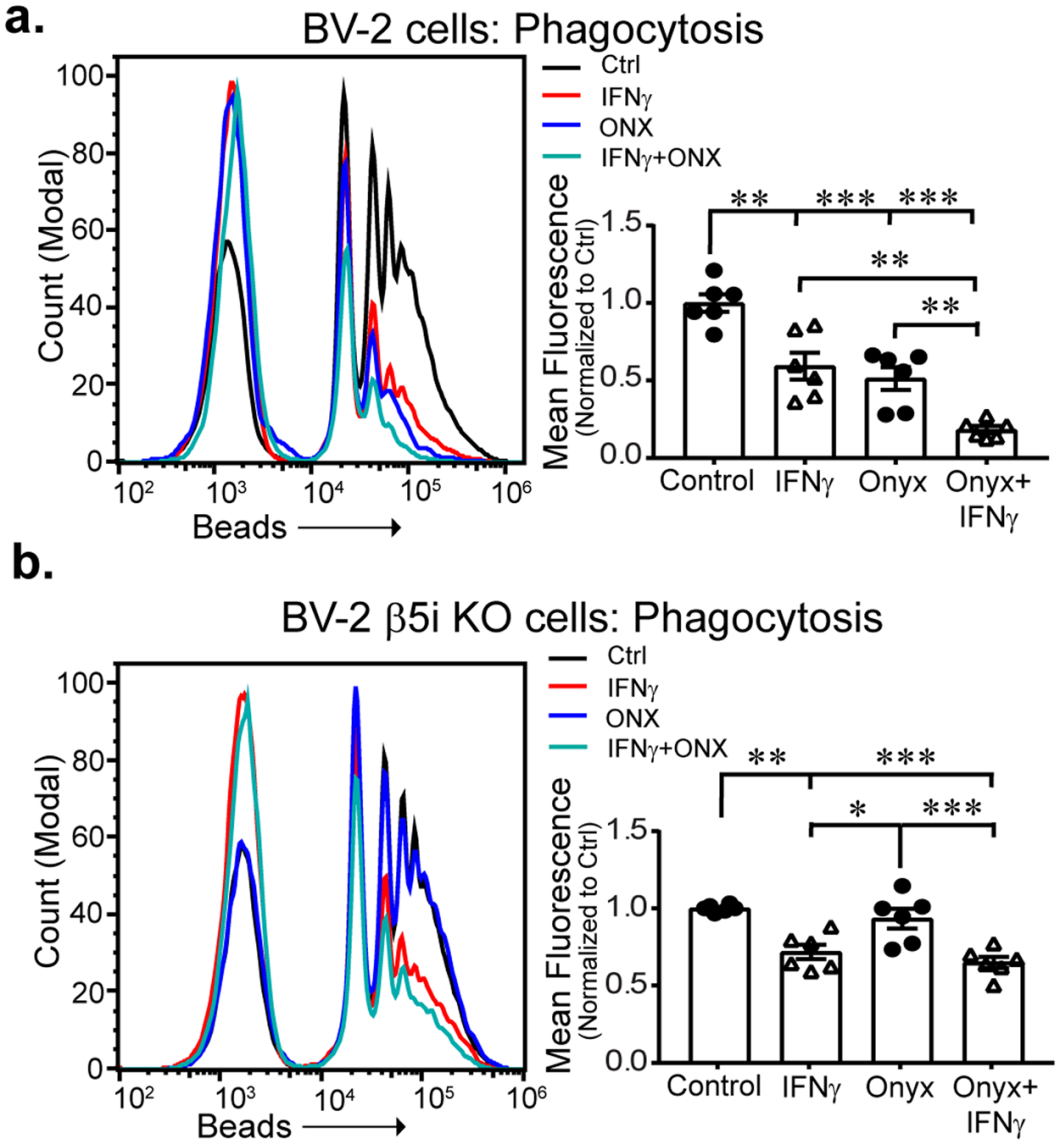
Immunoproteasome inhibition alters phagocytosis. (**a**) BV-2 cells were treated with IFNγ and/or ONX-0914 for 24 h. Treatment significantly alters phagocytosis of fluorescent microspheres (F(3,20) = 26.91, *p* > 0.001, n = 6, ANOVA with Tukey’s *post hoc*). Both IFNγ and ONX treatments significantly decrease phagocytosis (*p* = 0.001; *p* < 0.001, respectively). Co-treatment of IFNγ and ONX further decreases phagocytosis compared to control (*p* > 0.001), IFNγ (*p* = 0.001) and ONX (*p* = 0.008). (**b**) Phagocytosis in β5i KO BV-2 cells is different between groups (F(3,20) = 14.7, *p* < 0.001, n = 6). Mean fluorescence intensity is higher in control cells compared to IFNγ and ONX-0914/IFNγ co-treatment (*p* = 0.001 and *p* < 0.001, respectively). There was no difference between control and ONX-0914 treated cells.

A chief function of microglia *in vivo* is the removal of apoptotic cells, cell debris and pathogens by phagocytosis^38^. It has previously been shown that pro-inflammatory cytokines such as LPS, IL-1β, and IFNγ suppress microglial phagocytic activity *in vitro* ^39^. From our transcriptome analysis, we identified several genes known to function in phagocytosis including CD68, Spp1 and Mrc1 (CD206) that were reduced after IFNγ treatment and not restored by ONX-0914 co-treatment, suggesting that the immunoproteasome may not regulate IFNγ−dependent effects on phagocytosis. We used a flow cytometry bead based assay to investigate whether the phagocytic function of BV-2 cells was altered by the immunoproteasome. BV-2 cells incubated with fluorescent carboxylate-modified microspheres for 30 m produced an increase in fluorescence intensity (Fig. 7a; shift to the right), indicative of bead internalization. Cells treated with IFNγ displayed reduced MFI compared to untreated cells, indicative of reduced phagocytosis. Interestingly, ONX-0914 reduced MFI compared to untreated cells independent of IFNγ, suggesting they may act through different pathways. Consistent with this, we found that co-treatment of cells with IFNγ and ONX-0914 synergistically reduced phagocytosis compared to cells treated with IFNγ or ONX-0914 alone. Thus, IFNγ and ONX-0914 suppressed phagocytic activity of BV-2 cells, which was exacerbated when both drugs were given in combination. To confirm that the ONX-0914-dependent reduction of phagocytosis was not due to off-target drug effects, we performed the phagocytosis assay in BV-2 β5i KO cells (Fig. 7b). IFNγ partially reduced the phagocytic activity of BV-2 β5i KO cells compared to wild-type BV-2 cells, suggesting that the phagocytic activity was due, at least in part, by immunoproteasome activity. Moreover, ONX-0914 did not alter the phagocytic activity of BV-2 β5i KO cells observed with wild-type BV-2 cells (Fig. 7a). Therefore, the ONX-0914-dependent reduction in BV-2 phagocytic activity was mediated by the immunoproteasome, not off target drug effects. Together, these data suggest that the benefits to suppressing the early microglial pro-inflammatory response through inhibiting the immunoproteasome could be offset by subsequent inhibition of phagocytosis as microglia adopt an anti-inflammatory phenotype.

### Microglia priming is modulated by the immunoproteasome

**Figure 8:**
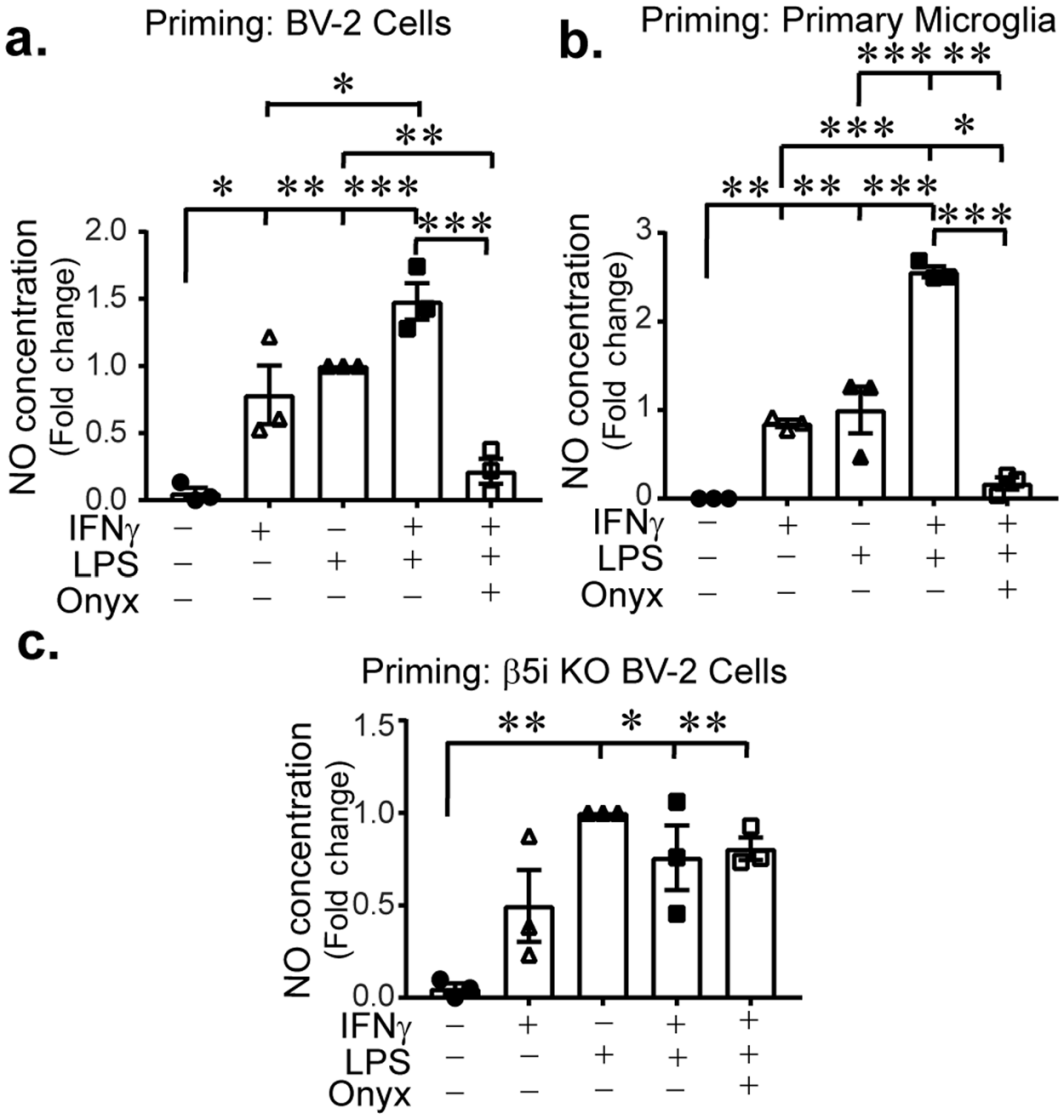
Microglia priming is mediated through the immunoproteasome. BV-2 or primary microglia were primed with IFNγ alone or in combination with ONX-0914 for 24 h. Following, cells were stimulated with LPS for an additional 24 h and NO levels were measured. (**a**) There is a significant effect of treatment on NO (F(4,10) = 22.183, *p* < 0.001, n = 3) in BV-2 cells. LPS induces NO production compared to control cells (*p* = 0.002), however IFNγ and LPS co-treatment results in exacerbated NO production (*p* < 0.001), indicative of the priming effect of IFNγ. Cells treated with ONX-0914 and IFNγ combined do not exhibit the priming response and NO is significantly lower than LPS + IFNγ (*p* < 0.001). There is no difference between control cells and IFNγ + ONX-0914 + LPS treated cells (*p* = 0.856). (**b**) NO release in primary microglia is significantly different between groups (F(4,10) = 64.44, *p* < 0.001, n = 3). IFNγ, LPS and IFNγ/LPS combination treatment elevates NO levels compared to control (*p* = 0.005, *p* = 0.001, *p* < 0.001, respectively). LPS resulted in an exaggerated NO response compared to LPS alone (*p* < 0.001), indicative of priming. Pre-treatment with ONX-0914 simultaneous with IFNγ priming, blocks the LPS-induced increase of NO, suggesting that immunoproteasome inhibition blocks priming. (**c**) β5i knockout BV-2 cells were treated in the same way as above, and NO was measured. There is a significant effect of treatment (F(4,10) = 9.454, *p* = 0.002, n = 3). LPS increased NO production compared to control (*p* = 0.002), however priming with IFNγ did not result in an LPS-induced increase of NO (*p* = 0.637). Data is presented as mean fold change normalized to LPS treatment.

Microglia priming has been suggested to occur during normal aging or by disruption of CNS homeostasis that transiently triggers release of inflammatory cytokines. Following exposure to IFNγ, microglia display an exaggerated inflammatory response to secondary insults resulting in enhanced reactivity and increased NO production (referred to as priming)^40^. To determine if the immunoproteasome plays a role in microglia priming, we treated BV-2 cells and primary microglia with IFNγ, or IFNγ and ONX-0914 for 24 h, then replaced the media with 0.1 µg/ml LPS. NO release was quantified after another 24 h. LPS alone resulted in production of NO, which was enhanced when cells were pre-treated with IFNγ (Fig. 8a). Interestingly, we found that when cells were co-treated with IFNγ and ONX-0914, NO levels returned to control levels, indicating that the immunoproteasome is required for boosting the LPS response. Primary microglia exhibited similar priming response as BV-2 cells, highlighting the usefulness of BV-2 cells for studying priming (Fig. 8b). Importantly, IFNγ pre-treated BV-2 β5i KO cells did not display enhanced NO production when subsequently treated with LPS, consistent with our pharmacological data (Fig. 8c). These data strongly suggest that IFNγ−dependent priming of microglia requires immunoproteasome activity.

## Discussion

The modular and dynamic composition of the proteasome and its multiple regulators allows the formation of various isoforms of the proteasome to fulfill a wide array of physiological functions. In the present study we begin characterizing the assembly of the immunoproteasome in microglia after 24 h of IFNγ treatment. Under normal conditions, most proteasomes exist as 26 S proteasome, however exposure to IFNγ, as well as oxidative and mitochondrial stress decrease 26 S and increase 20i proteasome levels^41–44^. IFNγ treatment results in the reduction of phosphorylation of two α subunits. By mutating serine residues 243 and 251 on Psma3, Bose and colleagues altered the stability of the 26 S proteasome and provided mechanistic evidence that IFNγ results in the loss of constitutive proteasome levels^41^. Data from the current study suggest that the switch from the constitutive to the immunoproteasome after exposure to IFNγ mediates aspects of the immune response by microglia. Moreover, the rapid immunoproteasome assembly requires the late stage proteasome maturation factor, Pomp. It remains unclear why this switch occurs since so far only subtle structural and functional differences between each proteasome subtype have been reported^45–49^. Presumably, the immunoproteasome confers additional or improved immune response functionality.

It is well known that the JAK/STAT pathway is activated by IFNγ and decreasing activation of JAK-STAT signaling can reduce inflammation in microglia^50^. Stat-1 (−/−) mice have decreased immunoproteasome levels, suggesting that basal levels of immunoproteasomes are regulated by the JAK/STAT pathway^51^. Given that JAK2/3 are the primary inflammatory signaling factor in hematopoietic cells, we hypothesized that JAK2/3 inhibition could decrease inflammation in microglia by modulating immunoproteasome levels. In this study, we provide direct evidence that inhibition of JAK2 and JAK3 reduce iNos gene expression and NO levels in BV-2 cells. Interestingly, JAK inhibition completely blocked immunoproteasome gene expression in response to IFNγ, suggesting immunoproteasome levels, and consequently the ratio of the different types of proteasomes, can be modulated through JAK signaling pathways.

Microglia respond to changes in the brain milieu by transitioning from a ramified to a reactive state^4^. Reactive microglia release pro-inflammatory cytokines, up-regulate MHC-I antigen presentation, and have increased pro-inflammatory cell surface receptors. Following stroke, microglia contain high amounts of immunoproteasomes and knockdown of immunoproteasomes results in decreased inflammation and improved recovery following ischemia in rats^14^. Generalized proteasome inhibitors have also been shown to decrease production of pro-inflammatory cytokines and improve recovery after TBI^15, 52–54^, however there is no evidence whether these effects are from inhibiting the constitutive proteasome, immunoproteasome or both. Here we provide evidence that ONX-0914, a selective immunoproteasome inhibitor, can reduce expression of various inflammatory markers in microglia. Treatment of neuroinflammation with immunoproteasome inhibitors rather than generalized proteasome inhibitors may provide a more viable therapeutic option with less adverse side effects.

There is considerable evidence that inflammation within the CNS increases with age^55, 56^ and contributes to age-related neurodegenerative diseases^57–61^. It has been shown in mouse models of prion disease and Alzheimer’s disease (AD) that pre-symptomatic challenge to the CNS markedly increased expression of IL-1β and iNOS during disease progression^62^. This suggests that primed microglia are more sensitive to secondary insults and display an exaggerated inflammatory response^63–65^. Thus, the age-dependent accumulation of molecules such as prion proteins and Aβ may trigger a chronic pro-inflammatory response that could be pathogenic or exacerbate the disease phenotype. Recent studies have revealed co-localization of microglia and protein aggregates in AD brains whereas others have suggested activated microglia as a clinical biomarker for AD^66^. Moreover, IFNγ levels are increased in Parkinson’s disease patients^67^ and IFNγ-deficient mice displayed attenuated 1-methyl-4-phenyl-1,2,3,6-tetrahydropyridine (MPTP)-induced substantia nigra pars compacta dopaminergic cell loss^68^ consistent with a role of IFNγ in neurodegeneration. In this study we show that exposure to IFNγ-primed microglia produced more NO after a second challenge. Importantly we show that IFNγ -dependent priming is suppressed by inhibiting the immunoproteasome signifying a potentially novel mechanism to suppress microglial priming. In addition to chronic CNS damage, immunoproteasomes may also modulate acute brain injuries. Indeed, as mentioned above, select inhibition of the immunoproteasome in a stroke model is sufficient to reduce inflammation and lesion volume^14^. These studies demonstrate that microglia are involved in both acute and progressive neuron damage and suggest that the immunoproteasome is, at least in part, responsible for mediating aspects of microglial activity.

**Figure 9:**
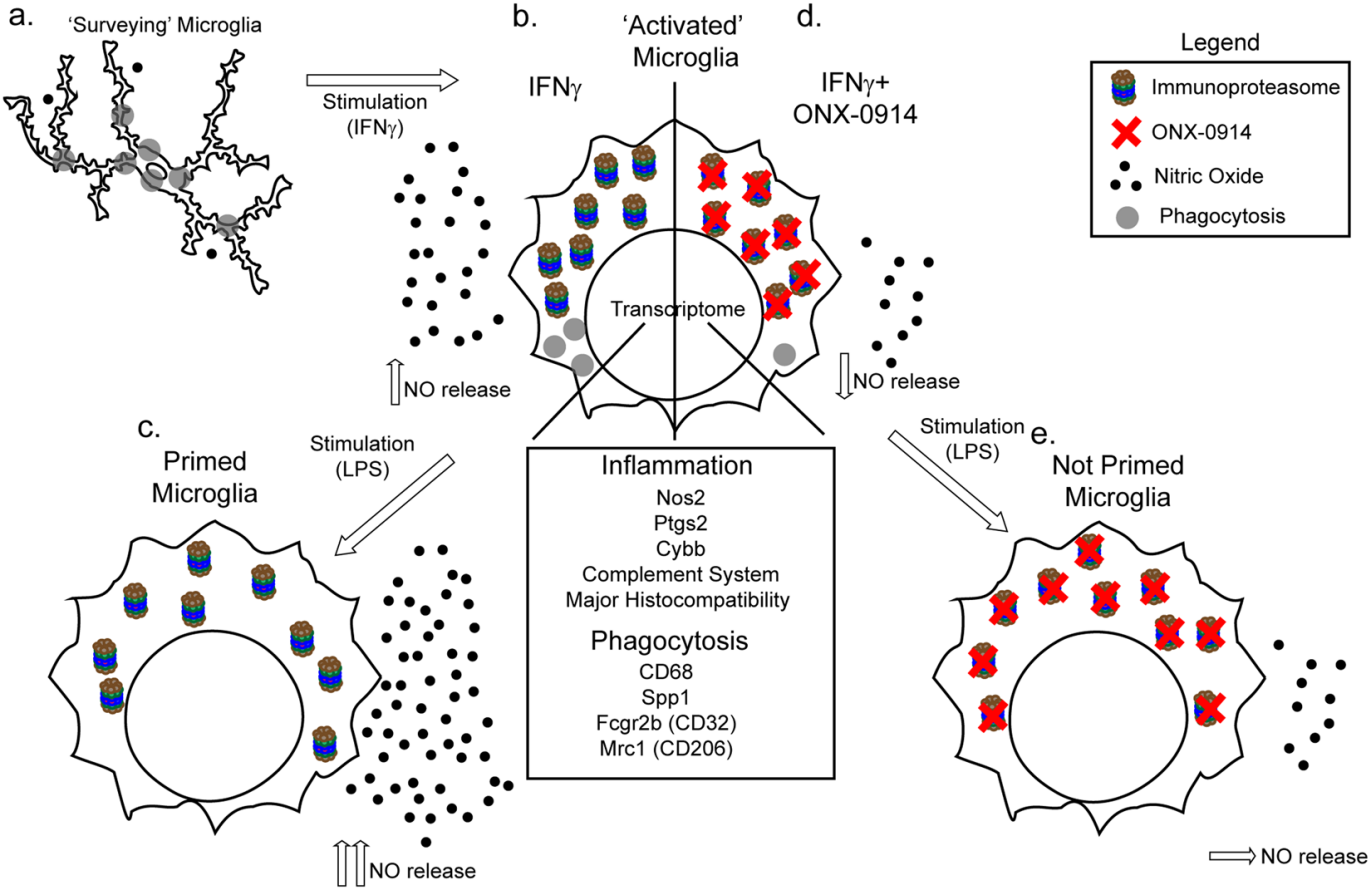
Immunoproteasome induction alters microglia function. Summary illustration of select microglia functions that are altered following ONX treatment. (**a**) Surveying microglia, in response to IFNγ, (**b**) transition to a reactive state accompanied by vast transcriptome alterations, up-regulation of the immunoproteasome, increased NO release and reduced phagocytosis. (**c**) IFNγ stimulated microglia become primed and respond more robustly to subsequent insults. (**d**) Immunoproteasome inhibition blocks the IFNγ-induced increase in NO release but exacerbates the reduction of phagocytosis. (**e**) Immunoproteasome inhibition protects cells from becoming primed, thus they do not have exaggerated response to a secondary stimulus.

In summary, we provide initial mechanistic characterization of the rapid formation of the immunoproteasome in microglia following exposure to IFNγ. We identified, using transcriptome profiling and validate *in vitro*, several features of microglial activation including NO production, phagocytosis and priming that require immunoproteasome activity (Fig. 9). Together our data indicate that targeting the immunoproteasome during or following trauma to the CNS will likely ameliorate microglia-mediated neuronal injury.

## Materials and Methods

All experiments were performed in accordance with approved guidelines, and all experimental protocols were approved in accordance with the regulations established by the Uniformed Services University of the Health Sciences.

### Animals

#### Controlled Cortical Impact Injury

Male C57Bl/6 J mice (Jackson Laboratories, Stock 000664, 12 weeks old) were subject to a left parietal lobe controlled cortical impact (CCI) injury (P-2.5 mm, L-1.5 mm, N = 13, (Dixon, Clifton *et al*. 1991, Smith, Soares *et al*. 1995)) or were sham prepared (N = 13). All procedures were approved by the Uniformed Services University Institutional Animal Care and Use Committee. Animals were housed in groups of 5 for the duration of the experiment with *ad libitum* access to water and food and maintained under 12 h light/dark cycle. Briefly, animals were anesthetized with gas Isoflurane (3% induction, 1–3% maintenance), shaved and scrubbed with 70% alcohol then Betadine. The level of anesthesia was assessed throughout the surgical procedure. Animals were placed in a stereotaxic frame (Kopf, Tujunga, California), then a midline incision was performed, skull and fascia retracted. At this point, animals randomly assigned to the sham group were sutured and allowed to recover in a heated chamber until they were fully awake. Injury animals were then given a craniotomy over the left parietal cortex (6 mm diameter). Care was taken to avoid damage to the cortex during the drilling process. The bone flap was removed and the cortex was impacted using the CCI device (Leica Microsystems, Buffalo Grove, IL, USA) with a sterile, circular impact tip (3 mm in diameter and 2.0 mm deep), to produce a severe injury at a velocity of 5 m/s. The scalp was then sutured and animals allowed to recover in a heated chamber until they were fully awake. The body temperature was maintained throughout the surgical procedure. Following the procedure, animals were returned to their home cage.

24 h following the CCI procedure, animals were deeply anesthetized with gas Isoflurane (3% induction, 1–3% maintenance), and were euthanized via cervical dislocation. Following, animals were decapitated and brains were extracted on dry ice and flash frozen in liquid nitrogen and stored at −80° C until analysis.

#### Tissue Preparation

Brains were removed from −80° C onto dry ice and the cortex at injury site was carefully excised from the brain tissue (0.05 g). Care was taken to eliminate the removal of tissue that was not part of the injury site. Tissue was homogenized in ice cold RIPA buffer (1% Triton X-100, 1% Sodium Deoxycholate, 150 mM NaCl, pH 7.5, 0.1% SDS, 1% Aprotinin, protease inhibitor). Tissue was spun down at 4° C for 15 m (14000 rpm, Eppendorf Centrifuge 5417 R). The supernatant was transferred to a 1.5 ml tube and was spun down again. The lysate was transferred to a new 1.5 ml tube and stored at −20° C. Before all biochemical experiments, a DC assay (500-0111, Bio-Rad, Hercules, CA, USA) was completed to determine protein concentration of all samples so that appropriate loading volume could be achieved. Each brain was prepared as 1n.

### Cell Culture

BV-2 cells were cultured under standard conditions in DMEM medium containing 5% FBS, 2 mM L-Glutamine, 100 units/ml penicillin and 100 μg/ml streptomycin. Cells were passaged 2–3 times per week.

Primary microglia were isolated from 2 day-old Sprague Dawley rat pups as previously described^69^. 8–10 neonatal pups were harvested into 8 cell culture flasks. After 10d, each flask was shaken for 1 h and all media/microglia was collected for 1 trial. Cells were maintained at 37°  C with 5% CO_2_ in media containing Dulbecco’s Modified Eagle Medium (Gibco), 1% L-glutamine (Gibco), 1% sodium pyruvate (Gibco), 10% FBS (Hyclone), and 1% penicillin and streptomycin (Thermo Fisher Scientific). Media was replaced every 3–5 days.

### β5i Knock-out BV-2 Cell Line

BV-2 cells were seeded to 70–80% confluency, then transfected with β5i double nickase plasmid containing GFP and Puromycin selection markers per manufacturer instructions (sc-421450-nic, Santa Cruz Biotechnology). 24 h following transfection, Puromycin (10 μg/ml) was added to the cells and maintained for 3 d. Following, cells were expanded in the presence of 2 μg/ml for 1–2 weeks and western blots were performed to validate the absence of β5i protein.

### Immunocytochemistry

To confirm colony purity, primary cells were fixed with 4% paraformaldehyde, and rinsed twice with 1x PBS. Primary antibodies included IBA-1 (Wako Life Sciences, #019-19741) and GFAP (abcam, ab4648). In addition, cell nuclei were stained with DAPI (Life Technologies, Carlsbad, CA). Fluorescently tagged secondary antibodies (Invitrogen) were visualized with an Olympus BX43 fluorescent microscope with a CellSens Standard imaging program. 5 images were randomly selected per sample. IBA-1 and GFAP positive cells were manually counted using NIH ImageJ.

### Transfections and siRNA knockdown

Plasmid transfection was performed using Lipofectamine 3000 (ThermoFisher) for 24–48 h according to the manufacturer protocols. siRNA knock-down of chaperone proteins (Pac1-50 nM, sense- GCAACGCAACAGCAUUCCUtt, anti-sense-AGGAAUGCUGUUGCGUUGCtt; Pomp-25 nM, Sense- CCUCUCGAGUUAUCAGAAAtt, Anti-sense- UUUCUGAUAACUCGAGAGGgt) was performed with RNAi Max (Life Technologies) using pre-validated siRNA from Ambion.

### Cytokine and Drug Treatment

BV-2 cells were plated 60–70% confluent and treated with 200 U/mL of IFNγ (R&D Systems) for 24–72 h. Primary microglia were plated at a density of 5 × 10^5^ cells/ml and treated with 1000 U/mL of recombinant rat IFNγ (R&D Systems). Immunoproteasome inhibition was achieved using 100 nM ONX-0914 (UBPBio) for 24 h. Jak3 inhibition was achieved using 1 μM of CP-690550 (InVivoGen) or JAk2 inhibition using AZD1480 simultaneous with IFNγ treatment. Priming experiments were performed by plating 1.0 × 10^4^ BV-2 cells or primary microglia (5 × 10^5^ cells/ml) in a clear 96-well plate. The following day, cells were treated in the following manner: 10 μl PBS, IFNγ, 100 nM ONX-0914 and IFNγ + 100 nM ONX-0914. 24 h later, media and treatments were removed and replaced with normal culture media in the presence or absence of 100 ng/mL LPS for an additional 24 h.

### RNA Isolation and Gene Expression Analysis

RNA was isolated using the Trizol and chloroform extraction method, then purified with the Qiagen RNeasy kit (#74104). cDNA conversion was performed using a Veriti thermal cycler and a high capacity cDNA conversion kit (Applied Biosystems). qRT-PCR was conducted with primers designed using Primer-Blast primer designing tool^70^, then obtained from Integrated DNA Technologies. All primers are listed in Supplemental Table S1. Psmb8 and NOS2 gene expression analysis was performed using pre-validated probes (Life Technologies). Gene expression of primary microglia was performed using pre-validated Taqman probes. Quantification occurred in a StepOnePlus qRT-PCR machine (Applied Biosystems) using SYBR Green RT-PCR master mix or Universal Probes master mix (BioRad) per manufacturer instructions.

### Transcriptome profiling by RNA sequencing

Total RNA was quantified via a fluorescence dye-based methodology (RiboGreen) on a Spectramax Gemini XPS plate reader (Molecular Devices, Mountain View, CA). RNA integrity was assessed using automated capillary electrophoresis on a Fragment Analyser (Advanced Analytical Technologies, Inc, Santa Clara, CA). Total RNA input of 200 ng was used for library preparation using the TruSeq Stranded mRNA Library Preparation Kit (Illumina, San Diego, CA). Sequencing libraries were quantified by PCR using KAPA Library Quantification Kit for NGS (Kapa, Wilmington, MA) and assessed for size distribution on a Fragment Analyser. Sequencing libraries were pooled and sequenced on a NextSeq. 500 Desktop Sequencer (Illumina) using a NextSeq. 500 High Output Kit v2 with paired-end reads at 75 bp length. Raw sequencing data was demuxed using bcl2fastq2 Conversion Software 2.17 before alignment using TopHat Alignment v1.0 and differential expression analysis using Cufflinks Assembly & DE v1.1.0 on BaseSpace Onsite (Illumina). Functional enrichment analysis was performed using the PANTHER Classification System^71^. Significantly overrepresented gene ontology biological processes at *p* < 0.01 were adjusted using a Bonferroni correction for multiple testing.

### Western and Native Gel Electrophoresis

For western blotting, cells were lysed (20 mM Hepes, 0.32 M Sucrose, 5 mM MgCl_2_, 2 mM ATP, 0.2% W/V NP-40, 2 mM DTT, Protease Inhibitor, pH 7.2), combined with Laemmli sample buffer, boiled for 5 m and 35 μg of protein was separated on BioRad Mini PROTEAN Bis-Tris gels. Native gel electrophoresis was conducted as described previously^72^. Briefly, cells were lysed and combined with native sample buffer and G250 (Invitrogen). Equal amounts of sample were separated in 4–16% gradient Bis-Tris Invitrogen native page gels at 4 C for 1 h at 150 V then increased to 200 V for an additional h. Following, proteins were transferred to poly(vinylidene difluoride) membranes using a Transblot Turbo. All antibodies were obtained from Abcam unless otherwise stated: Psma3 (ab109532), Psmb5 (ab3330), Psmb8 (ab3329), Psmg1 (ab167396), POMP (D2 × 9 S, Santa Cruz), β actin (A3854, Sigma).

### Nitric Oxide Assay

The Griess Reagent System (Promega) was used to measure nitrite, one of the stable breakdown products of NO, per manufacturer instructions. Briefly, 24 h following treatment, cell culture media was transferred to a new 96-well plate. Sulfanilamide was added to the media and incubated in the dark for 10 m. Next, *N-* 1- napthylethylenediamine dihydrochloride (NED) was added and incubated in the dark for 10 m. Plate was then read between 520 nm and 550 nm. All experiments were performed in technical triplicates. Absorbance values were then compared to a nitrite standard curve and normalized.

### Phagocytosis Assay

Red (Ex580/Em605) carboxylate-modified microspheres (FluoSpheres, F8821, ThermoFisher Scientific) were obtained and prepared as previously described^73^. Briefly, FluoSpheres were pelleted at 10,000 g for 15 m at room temperature. Supernatant was removed and the pellet was washed in pure water and pelleted again. Following, water was removed and FluoSpheres were re-suspended (3% BSA, 25 mM Na_2_HPO_4_, pH 6.0) by rapid vortexing.

3 × 10^5^ BV-2 cells were plated in a 6-well plate. 24 h later, cells were treated with IFNγ, ONX-0914 or both for an additional 24 h. Following, 15 μL of beads were added directly to each well for 30 m at 37° C. To collect cells for analysis, media was removed and cells were washed twice with PBS, then gently scraped and centrifuged at 300 g for 10 m. Cell pellets were re-suspended in FACS buffer and submitted to flow cytometry (BD Accuri C6, BD Biosciences). Cells were gated so that only viable cells were counted. 10,000 cells/treatment group were counted. To adjust for background, control cells that did not have beads were used for each experiment (n = 6). The mean fluorescence of all viable cells was measured and data were analysed using an ANOVA with Tukey’s *post hoc* analysis.

### Flow Cytometry

Following treatment, media was removed and cells were washed with warm PBS. Cells were then gently dislodged from the plate by scraping and pelleted by centrifugation. Cells were re-suspended in FACS buffer and incubated with CD40 antibody (4 μl antibody/100ul FACS buffer, PE anti-mouse CD40, clone 3/23, #124609, Biolegend) on ice for 20 m. Cells were then washed with FACS buffer twice and read in the PE channel (BD Accuri C6). Mean fluorescence intensity was recorded.

### Cell extract proteasome activity

To determine changes in proteasome activity, a fluorogenic substrate assay was performed. BV-2 cells were lysed in buffer (20 mM Hepes, 0.32 M Sucrose, 5 mM MgCl_2_, 2 mM ATP, 2 mM DTT, protease and phosphatase inhibitors, pH 7.2^74^). The supernatant was utilized to measure Chymotryptic-like (Suc-Leu-Leu-Val-Tyr-AMC, S280, Boston Biochem), peptidyl glutamyl peptide-hydrolyzing (PGPH, Z-Leu-Leu-Glu-AMC, S-230, Boston Biochem) and trypsin-like (Ac-Arg-Leu-Arg-AMC, S-290, Boston Biochem) activities. After protein concentration of the individual samples was acquired, 75 µg of tissue was combined with 20 S reaction buffer (20 mM Tris-Cl, 1 mM EDTA, 1 mMNaN_3_, 1mM DTT, pH 7.5) to a volume of 100 µl and was added to a black, clear bottom 96 well plate. 20 µL of Suc-Leu-Leu fluorescent substrate buffer (100 mM with 20 S reaction buffer) was added to the samples in the well plate. All experiments were conducted in duplicates. The plate was then read at 355ex/460em at 37 C with a FLUOROstar Omega plate reader. Data was collected immediately after substrate addition and continued for 30 m. All data was automatically corrected based on blank wells that contained only the fluorescent substrate.

### In cell Proteasome Activity

1 × 10^4^ BV-2 cells were plated in a clear bottom, black 96-well plate. 24 h following, they were treated with IFNγ in the absence and presence of MG-132 (10 μM, Enzo Life Sciences) for 24 h then subject to a live cell chymotrypsin-like proteasome activity assay (Proteasome-Glo, Promega) per manufacturer instructions.

### Data Analysis

All data were analysed using SPSS24 for Windows. Outliers were removed by calculating the Z-score (95% confidence interval) for each data set. Data points where Z > 2 were considered outliers and omitted from analysis. For analysis between 2 groups (Control and IFNγ, sham and injury) an independent samples t-test (2-tailed) was conducted. Levene’s test was used to assess variance and normality and appropriate transformations were applied when necessary. For experiments involving multiple treatment groups, one-way analysis of variance was used (ANOVA), with Tukey’s HSD *post-hoc* to compare differences between groups when appropriate. For all figures, *p < 0.05, **p < 0.01, ***p < 0.001. Data are presented as mean values ± standard error of the mean (SEM).

### Data availability

The data that support the findings of this study are available from the corresponding author upon reasonable request.

## Electronic supplementary material

Supplementary Figures
