# Supplementary material for: The role of the immunoproteasome in interferon-γ-mediated microglial activation
Source: Sci Rep. 2017 Aug 24;7:9365. doi: 10.1038/s41598-017-09715-y (PMC5571106; doi:10.1038/s41598-017-09715-y)
Supplement: Supplementary file 1 — Supplementary Figures [file 41598_2017_9715_MOESM1_ESM.pdf]

Title: **The role of the immunoproteasome in interferon-γ-mediated microglial activation**

Kasey E. Moritz[1], Nikki M. McCormack[1], Mahlet B. Abera[2], Coralie Viollet[3], Young J. Yauger[1], Gauthaman Sukumar[3], Clifton L. Dalgard[1,2,3,4], and Barrington G. Burnett[1,2*]

[1]Neuroscience Program, Uniformed Services University of the Health Sciences, F. Edward Hebert School of Medicine, Bethesda, MD, USA.

[2]Department of Anatomy, Physiology and Genetics, Uniformed Services University of the Health Sciences, Bethesda, MD, USA.

[3]Collaborative Health Initiative Research Program, Uniformed Services University of the Health Sciences, Bethesda, MD, USA.

[4]The American Genome Center, Uniformed Services University of the Health Sciences, Bethesda, MD, USA.

**Corresponding author*: Barrington G. Burnett, 4301 Jones Bridge Rd, Bethesda, MD 20814, Email: bburnett@usuhs.edu, Tel: (1) 301 295 3506, Fax: (1) 301 480 3365

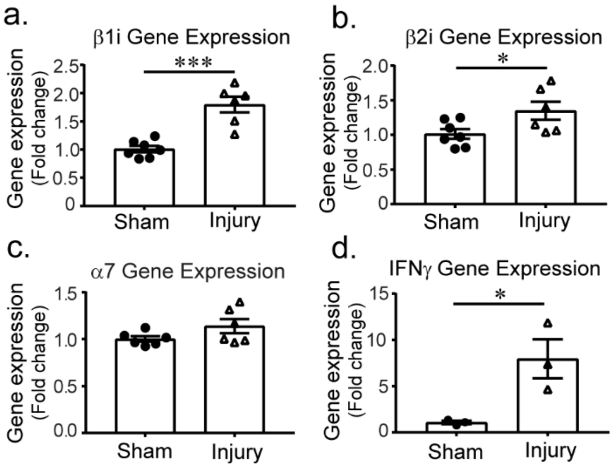

Supplemental Figure 1. TBI-induced gene expression changes. a. Immunoproteasome subunits psmb9 (β1i, t(11)=5.564, *p*<.001)) and **b.** Psmb10 (β2i, t(11)=2.367, *p*=.037)) gene expression is increased 24 h following TBI (n=7). **c.** TBI does not alter all proteasome subunits, constitutive proteasome subunit α7 is unchanged (t(10)=1.695, *p*=.121, t-test). **d.** TBI induces IFNγ gene expression (t(4)=-3.264, *p*=.031, n=3, t-test).

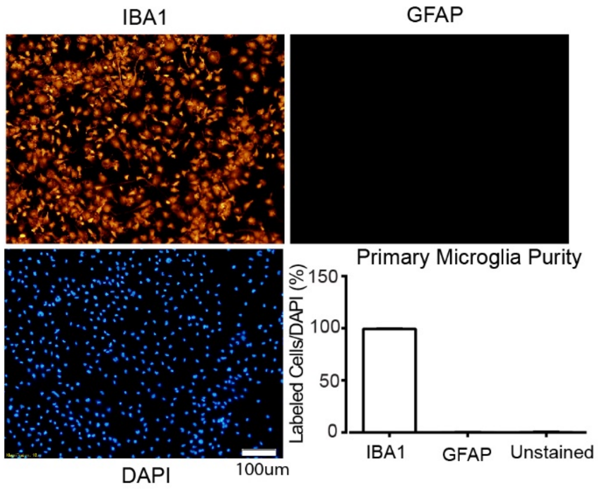

Supplemental Figure 2: Primary microglia purity. Primary microglia were isolated and purity is confirmed to be >98%. (IBA1-Ionized Calcium Binding Adaptor Molecule 1; DAPI-4',6-Diamidino-2-Phenylindole, Dihydrochloride; GFAP- Glial Fibrillary acidic protein).

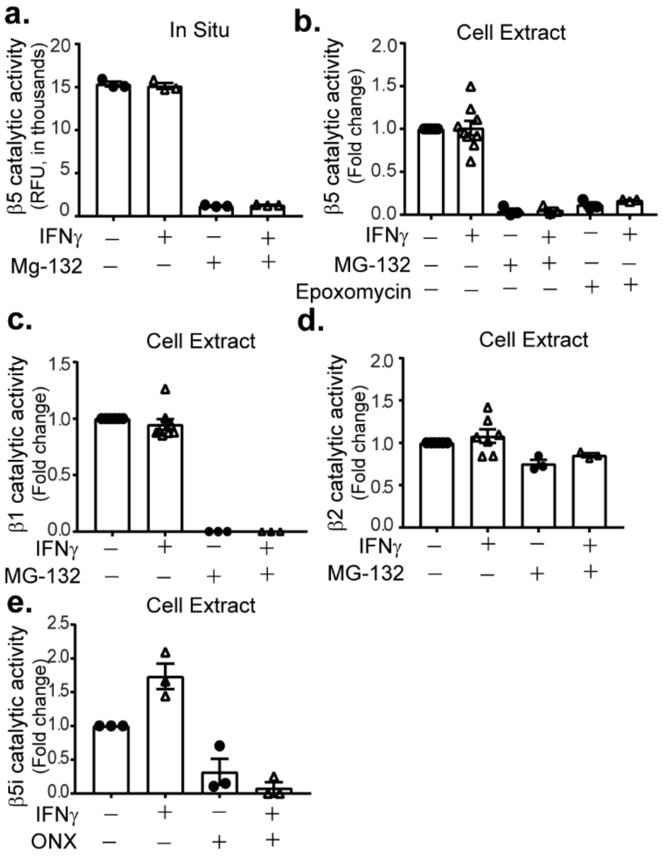

Supplemental Figure 3: Proteasome catalytic activity following IFNγ treatment. a. Chymotrypsin-like activity of β5 subunit was measured in live cells in the absence and presence of IFNγ. There is no change in proteolytic cleavage of the fluorescent substrate ($t(4)=.484$, $p=.654$, n=3). **b**. β5 proteasome activity was also measured in cell extracts and is different between treatment groups ($F(5,24)=55.17$, $p<.001$). Analyses confirmed that IFNγ treatment does not alter activity compared to control (n=9). **c.** Caspase-like β1 activity is significantly different following treatment ($F(3,18)=192$, $p<.001$). There is no difference between control and IFNγ treated groups (n=6). **d.** Trypsin-like catalytic activity of β1 subunit was measured in cell extracts and was determined to be different between groups ($F(3,17)=5.472$, $p=.008$). No difference observed between control and IFNγ treated groups. **e.** β5i immunoproteasome activity was measured in the absence and presence of IFNγ and immunoproteasome inhibitor. There is a significant treatment effect ($F(3,8)=27.47$, $p<.001$, n=3). β5i activity is significantly higher following IFNγ treatment compared to control ($p=.026$) and IFNγ and ONX-0914 co-treatment ($p<.001$).

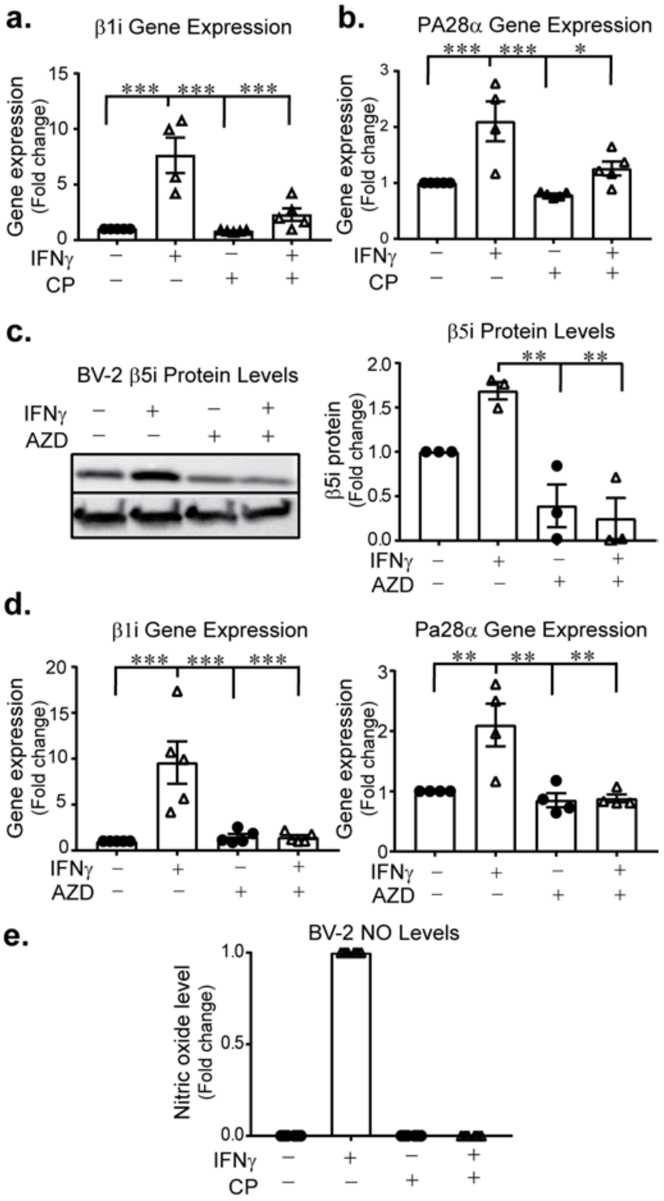

Supplemental Figure 4: Jak inhibition modulates IFNγ-induced immunoproteasome gene expression. a. Treatment significantly impacts β1i gene expression (F(3,15)=17.755, p<.001). 24 h IFNγ (n=4) treatment increases β1i levels compared to control (n=5, p<.001), CP-690550 (n=5, p<.001) and IFNγ+CP-690550 co-treatment (n=5, p=.001). There was no difference between control and IFNγ+CP-690550 co-treatment (p=.571). **b.** PA28α gene expression is significantly different (F(3,15)=11.642, p=.001). IFNγ increases gene expression compared to control (p=.001), CP-690550 (p<.001) and IFNγ+CP-690550 co-treatment (p=.013). **c.** Treatment of BV-2 cells with Jak1/2 inhibitor, AZD1480 (AZD, 1 μM, blocks IFNγ–induced immunoproteasome β5i protein increases (F(3,8)=14.208, p=.001, n=3 ; IFNγ vs IFNγ+AZD,

p=.002). **d.** Gene expression of immunoproteasome subunit β1i is significantly different after IFNγ and AZD1480 treatment ($F(3,16)=12.568$, $p<.001$, n=5). IFNγ significantly increases gene expression compared to control ($p<.001$) and AZD1480 co-treatment blocks the increase ($p=.001$). PA28α gene expression is also altered by IFNγ and/or AZD1480 treatment ($F(3,12)=9.963$, $p=.001$, n=4, one-way ANOVA, Tukey's post-hoc,). Expression is increased by IFNγ ($p=.007$), however co-treatment with AZD blocks the IFNγ-induced up-regulation ($p=.003$). **e.** NO levels are increased by IFNγ treatment, however are undetectable in cells treated with CP-690550.

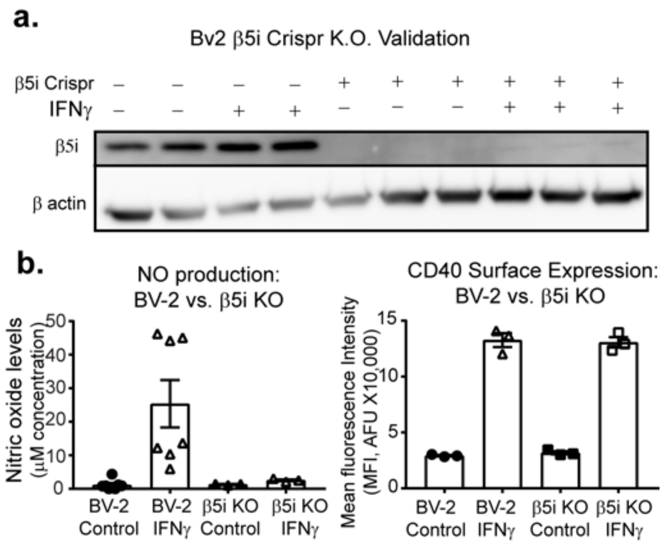

Supplemental Figure 5: CRISPR/CAS9n mediated Psmb8 K.O. Validation. a. To validate knockout of β5i in BV-2 cells, normal and knock-out BV-2 cells were treated with IFNγ for 24 h to induce the immunoproteasome. Western blot analysis confirmed that no detectable β5i is present. **b.** BV-2 cells lacking the β5i subunit have an altered NO response to IFNγ compared to normal BV-2 cells ($F(3,16)=6.579$, $p=.004$, BV-2 n=7, KO n=3). IFNγ significantly increased NO release in BV-2 cells ($p=.005$), but not in β5i KO cells. **c.** Flow cytometry analysis was performed to confirm that BV-2 β5i KO cells are stimulated by IFNγ. There is a significant difference between treatment groups ($F(3,8)=216.3$, $p<.001$, n=3). IFNγ treatment results in a significant increase of CD40 cell surface expression in both normal BV-2 cells ($p<.001$) and β5i KO cells ($p<.001$), however there are no differences between normal and β5i KO BV-2 cells.

Supplementary Table 1: List of genes and associated primers used for qRT-PCR gene expression analysis.

Gene	Forward (5'-3')	Reverse (5'-3')
**Proteasome Subunits**		
**Psma1**	GCAACTTTATGCGCCAGGAG	AGGCACGCAGACCATGTTTA
**Psma2**	GTGTTCCGACTTTCCGGGTA	TACCGCAGCCAAGGCATATT
**Psma3**	GCGCTGTTAGACCTTTTGGC	ACATCACGGCAAGTCATTTCC
**Psma4**	CTCACCGTCTTCCTCTGGAATCA	AAAATCCCCAAACAGGTGCC
**Psma5**	AGTGCTCTCTAGTTCCGCCT	GGCCACACGATTGAAAGCAG
**Psma6**	TCCCTGGTGTGTGTGCTACG	GAGTGGCCTCATTTCAGCGT
**Psma7**	AAAAATCTGCGCCTTGGACG	GCCGAGATACCAAATGGCCT
**Psmb1**	CTGCTTACCGAGACGTGGAA	AGTGTCTGAAGCGACGATGG
**Psmb2**	TCCTATGTGTTGGAGAGGCT	AAGTTCCCTGGTGAGTGGGA
**Psmb3**	GTGTGAGTCTCTCTGGGAGC	AGCACAGGTTAGTCCATCCG
**Psmb4**	TGGAAGCGTTTTGGGAGTCA	CTTCACCCCGAGTACCGATG
**Psmb5**	CTCTGGCTCCGTGTATGCTT	ACATTGTCACTGGACACCCG
**Psmb6**	GGCAGGTGTACTCTGTTCCC	CTTGGTCATGCCTTCCCGAT
**Psmb7**	ACTTGGGGTCTGGAAGCAAC	GACAGCGGTAGTGCCTTTCT
**Psmb9**	GAAGAAGTCCACACCGGGAC	TGGTCCCAGCCAGCTACTAT
**Psmb10**	CAGAGAGAAACACGTGACAGACTGG	CAGGACAGGTGTGGTTCCAGGAGC
**Psmc6**	ATCATGGCGGACCCTAGAGA	GACGGCCACCAATAGCATCT
**Psmd4**	AATCGGAGGTGCTGTTGTTT	GCCCACGTTATTCTCAGGGT
**Psmd11**	GTGAGAGCGGTAAGATGGCG	TTTGCTGTGGTTCGAGCAGA
**Psme1**	ATTGTGGTCCTCCTGCAACG	ATCTCCTGGTACTCCGCCTC
**Proteasome Chaperones**		
**Psmg1**	GCAGTAGGAAGCAACGCAAC	TCTGGGTTGGAAGCCGAAAA
**Psmg2**	CTCCTGATGCCAGCAGTATCC	CAGGGATGCACCGACTCTTT
**Psmg3**	AGTAGTGATCCAGCCATCCCC	GCGACACAAATGCTACCAGG
**Psmg4**	GCGCGCATCTGAAAGTGAAA	CACCCACAGGAAGAGCGAAT
**Pomp**	AGTGAACTGATGGGTGAGCC	TCTCAACTGACCCACAACCC

Supplementary Table 2: Proteasome subunit relative expression													Average		
**Gene**	Control1	Control2	Control3	IFN1	IFN2	IFN3	ONX1	ONX2	ONX3	IFN+ONX1	IFN+ONX2	IFN+ONX3	control	IFN	ONX	IFN + ONX
**Psma1**	138.2	141.7	145.88	125.62	119.16	126.44	206.16	226.03	198.19	264.97	228.95	245.78	141.9267	123.74	210.1267	246.5667
**Psma2**	230.82	245.3	240.22	210.59	193.37	221.16	325.13	358.18	295.89	377.61	348.99	353.38	238.78	208.3733	326.4	359.9933
**Psma3**	164.31	157.82	160.86	311.79	300.16	324.57	198.93	228.01	192.54	301.85	318.76	305.13	160.9967	312.1733	206.4933	308.58
**Psma4**	157.43	154.88	159.33	162.71	161.19	168.3	208.7	223.58	193.28	254.5	212.81	233.55	157.2133	164.0667	208.52	233.62
**Psma5**	238.47	226.38	233.32	295.41	287.64	317.31	364.97	396.42	342.55	656.59	552.8	587.26	232.7233	300.12	367.98	598.8833
**Psma6**	196.34	193.76	210.02	160.11	157.63	175.84	222.29	251.98	211.74	251.71	233.2	239.15	200.04	164.5267	228.67	241.3533
**Psma7**	324.58	330.01	328.06	413.41	393.41	470.43	424.75	498.19	425.2	657.59	648.55	644.99	327.55	425.75	449.38	650.3767
**Psma8**	0	0	0	0	0	0	0	0	0	0	0	0	0	0	0	0
**Psmb1**	145.5	150.25	141.51	140.24	132.28	147.58	195.59	218.14	184.56	240.78	227.22	227.69	145.7533	140.0333	199.43	231.8967
**Psmb10**	86.65	86.42	80.52	322.93	302.25	337.82	67.2	77.65	69.15	249.72	244.74	251.23	84.53	321	71.33333	248.5633
**Psmb11**	0	0	0	0	0	0	0	0.01	0	0.01	0.04	0.02	0	0	0.003333	0.023333
**Psmb2**	169.79	179.78	175	163.55	156.22	172.36	202.82	227.45	197.24	267.17	254.11	250.39	174.8567	164.0433	209.17	257.2233
**Psmb3**	251.16	250.41	244.11	257.29	248.19	277.66	389.24	428.04	353.21	548.44	476.87	515.86	248.56	261.0467	390.1633	513.7233
**Psmb4**	360.01	353.2	347.83	386.06	358.38	403.56	532.09	613.05	473.69	709.76	639.72	664.68	353.68	382.6667	539.61	671.3867
**Psmb5**	409.37	409.8	382.4	355.39	341.57	392.24	529.94	595.24	485.19	593.3	572.15	603.82	400.5233	363.0667	536.79	589.7567
**Psmb6**	263.55	265.58	243.34	296.59	273.22	291.57	353.9	372.91	305.64	511.55	459.53	492.49	257.49	287.1267	344.15	487.8567
**Psmb7**	315.05	302.18	312.42	329.26	346.35	361.3	421.09	473.55	399.77	558.09	539.36	565.02	309.8833	345.6367	431.47	554.1567
**Psmb8**	85.36	86.68	76.13	340.07	333.55	359.79	75.56	85.9	76.02	248.07	276.43	277.49	82.72333	344.47	79.16	267.33
**Psmb9**	27.21	29.6	24.53	253.63	235.61	266	26.81	27.99	23.34	161.39	185.81	173.97	27.11333	251.7467	26.04667	173.7233
**Psmc1**	161.09	147.96	155.75	114.32	114.24	116.86	208.03	216.73	188.62	257.04	220.37	239.74	154.9333	115.14	204.46	239.05
**Psmc2**	100.85	94.22	96.41	82.96	81.58	88.84	150.3	160.42	138.79	206.91	186.82	199.26	97.16	84.46	149.8367	197.6633
**Psmc3**	164.29	152.63	148.95	133.91	138.85	151.86	240.55	251.04	205.28	370.92	312.9	365.27	155.29	141.54	232.29	349.6967
**Psmc3ip**	11.69	12.4	12.76	10.3	12.88	17.12	15.24	14.16	12.04	13.01	10.34	14.32	12.28333	13.43333	13.81333	12.55667
**Psmc4**	97.12	87.82	86.05	66.96	66.07	71.63	125.14	132.61	108.2	212.23	166.83	187.84	90.33	68.22	121.9833	188.9667
**Psmc5**	148.8	136.87	139.98	118.46	112.37	118.67	196.25	213.07	188.47	254.45	227.23	249.1	141.8833	116.5	199.2633	243.5933
**Psmc6**	148.08	143.35	157.39	123.79	132.03	134.56	214.23	228.19	208.25	373.13	318.8	351.98	149.6067	130.1267	216.89	347.97
**Psmd1**	65.76	62.26	62.23	48.59	48.63	45.86	98.84	96.69	94.74	132.63	117.18	128.57	63.41667	47.69333	96.75667	126.1267
**Psmd10**	52.18	49.49	50.12	63.14	54.68	58.11	60.43	61.82	51.5	69.7	66.47	64.25	50.59667	58.64333	57.91667	66.80667

**Psmd11**	76.37	66.12	73.22	63.27	61.2	57.08	107.8	100.89	97.6	186.13	140.67	163.64	71.90333	60.51667	102.0967	163.48
**Psmd12**	64.77	66.11	65.8	49.29	50.84	50.7	79.6	86.42	77.64	101.46	88.41	100.31	65.56	50.27667	81.22	96.72667
**Psmd13**	72.09	72.76	71.34	57.76	61.01	58.67	87.01	95.33	84.98	118.57	101.77	110.24	72.06333	59.14667	89.10667	110.1933
**Psmd14**	104.67	102.31	107.16	90.09	89.51	96.29	126.68	138.86	127	186.72	158.53	175.36	104.7133	91.96333	130.8467	173.5367
**Psmd2**	183.98	178.72	182.74	147.4	156.66	149.13	275.59	256.05	249.97	316.96	277.45	307.33	181.8133	151.0633	260.5367	300.58
**Psmd3**	83.47	76.41	79.92	79.79	79.08	80.16	113.64	112.32	100.62	158.29	147.75	155.48	79.93333	79.67667	108.86	153.84
**Psmd4**	180.59	161.91	161.52	157.11	149.16	168.62	276.37	299.19	245.77	431.57	400.6	423.54	168.0067	158.2967	273.7767	418.57
**Psmd5**	33.79	36.51	36.71	38.14	33.66	35.11	42.98	43.08	39.37	67.88	60.68	69.86	35.67	35.63667	41.81	66.14
**Psmd6**	74.21	73.23	69.96	55.28	51.38	52.66	87.16	99.37	85.23	120.09	108.93	114.96	72.46667	53.10667	90.58667	114.66
**Psmd7**	135.08	132.15	132.97	118.13	115.37	121.24	175.39	195.59	172.05	212.68	185.41	201.15	133.4	118.2467	181.01	199.7467
**Psmd8**	162.53	183.33	177.51	192.77	179.29	200.97	171.77	194.68	172.27	212.28	230.48	216.55	174.4567	191.01	179.5733	219.77
**Psmd9**	13.81	13.97	13.35	11.51	11.52	13.92	14.38	16.33	13.88	17.71	16.05	17.07	13.71	12.31667	14.86333	16.94333
**Psme1**	181.36	202.91	170.1	648.28	664.06	767.29	187.23	221.7	176.95	643.28	675.17	696.94	184.79	693.21	195.2933	671.7967
**Psme2**	206.39	216.91	205.77	701.82	652.07	772.36	189.8	222.1	184.6	576.73	582.55	588.5	209.69	708.75	198.8333	582.5933
**Psme2b**	11.67	13.4	10.85	44.24	41.29	43.7	9.87	11.76	11.24	42.87	43.63	39.67	11.97333	43.07667	10.95667	42.05667
**Psme3**	77.42	76.09	77.26	55.53	54.8	57.77	73.56	70.12	71.14	65.55	56.24	61.6	76.92333	56.03333	71.60667	61.13
**Psme4**	22.87	22.33	24.03	22.16	22.99	20.1	28.97	25.55	27.62	40.11	36.03	39.2	23.07667	21.75	27.38	38.44667
**Psmf1**	12.11	12.5	13.26	21.53	20.59	23.03	14.64	14.07	13.22	35.28	40.04	38.1	12.62333	21.71667	13.97667	37.80667
**Psmg1**	59.35	63.65	64.6	42.59	45.41	48.33	53.16	64.4	57.58	40.28	41.91	44.24	62.53333	45.44333	58.38	42.14333
**Psmg2**	60.76	62.59	63.48	61.89	58.96	66.25	54.75	64.39	60.02	52.37	48.83	56.7	62.27667	62.36667	59.72	52.63333
**Psmg3**	50.37	50.81	49.8	42.1	39.44	48.91	46.41	62.89	44.52	46.89	44.29	53.41	50.32667	43.48333	51.27333	48.19667
**Psmg4**	32.15	29.78	36.18	46.42	40.93	41.73	21.22	29.69	28.58	30.74	33.26	32.09	32.70333	43.02667	26.49667	32.03

Supplementary Table 3. List of 703 Genes Altered by Interferon-gamma and reversed by ONX-0914

There are 588 genes up-regulated with IFN and down-regulated with ONX-0914; and there are 115 genes down-regulated with IFN and up-regulated with ONX-0914

**IFN-γ**	**Up-regulated**	**Down-regulated**
**IFN-γ + ONX-0914**	**Down-regulated**	**Up-regulated**
	0610040F04Rik	1110019D14Rik
	1700001K19Rik	1700001L05Rik
	1700008I05Rik	1700102H20Rik
	1700013F07Rik	1810006J02Rik
	1700019O17Rik	2610203C22Rik
	1700025N23Rik	4930451G09Rik
	1700028P14Rik	4930479D17Rik
	1700096K18Rik	9530052E02Rik
	1700101E01Rik	A930001C03Rik
	1700113A16Rik	A930019D19Rik
	1700123M08Rik	AK019690
	2010002M12Rik	AK033433
	2010005H15Rik	AK036547
	2010109I03Rik	AK037411
	2410003L11Rik	AK045483
	2410022M11Rik	AK049499
	2900005J15Rik	AK080292
	4632428C04Rik	AK085138
	4921529L05Rik	AK140018
	4930506C21Rik	AK145533
	4930512M02Rik	AK153821
	4930539E08Rik	AK163440
	4930539M17Rik	AK165297
	4930555B11Rik	AK196308
	4933409K07Rik	AK202488
	4933433G15Rik	AK203921
	5430402O13Rik	Adcy3
	5730508B09Rik	Afp
	5830428M24Rik	Alox15
	6330403A02Rik	Ankrd34a
	9130230L23Rik	BC060616
	9230116N13Rik	BC127131
	9330182L06Rik	Camk4
	9530003J23Rik	Car14
	9530062K07Rik	Ccdc73
	A330074K22Rik	Ccne2

	A530050N04Rik	Cd151
	A630023A22Rik	Cdcp1
	A930003O13Rik	Cdhr3
	AB346163	Ceacam1
	AI182371	Ceacam2
	AI413582	Cldn15
	AK004434	Col4a1
	AK005616	Cox20
	AK006576	Daam2
	AK007907	Dlst
	AK009067	Ermap
	AK009639	Fam83c
	AK012238	Fkbp14
	AK013187	Frk
	AK015529	Gdpd2
	AK015772	Gm12359
	AK017143	Gm14295
	AK018679	Gm14391
	AK019679	Gm14440
	AK031925	Gm15446
	AK034260	Gm15915
	AK035387	Gm19434
	AK038969	Gm19510
	AK039256	Gp9
	AK039820	Gstm1
	AK040967	Hexim1
	AK042791	Igfbp2
	AK044033	Kcnc1
	AK044300	Kcnh2
	AK044689	Klhl3
	AK045867	Lhx6
	AK047541	Lipm
	AK048349	Lix1
	AK048886	Lmbr1
	AK050290	Lrrc36
	AK052070	Lrrc39
	AK076358	Lyve1
	AK076906	Mdk
	AK077348	Msrb2
	AK077832	Mustn1
	AK079912	Myc

	AK084133	Myl2
	AK084733	Mylpf
	AK085384	Ngb
	AK085768	Nkpd1
	AK087099	Npas1
	AK087755	Nt5c2
	AK087850	Nwd1
	AK089217	Pianp
	AK089519	Pik3r3
	AK131831	Plcb4
	AK132074	Plin2
	AK134546	Pnp2
	AK135262	Prr33
	AK135406	Rph3a
	AK137190	Rpl3l
	AK137643	S100a4
	AK138068	Serpinf2
	AK138088	Slc16a12
	AK138323	Slc25a47
	AK138451	Slc26a8
	AK138921	Slc39a12
	AK139076	Slc5a5
	AK143184	Snora26
	AK146694	Sox15
	AK148054	Spats1
	AK149321	Srrt
	AK155239	Star
	AK156859	Supt4a
	AK158473	Tbata
	AK158920	Tet1
	AK159995	Trim15
	AK161019	Trim38
	AK162078	Trpv4
	AK162363	Tubb4b
	AK163103	Wwc1
	AK165223	X57780
	AK171153	Zfp385c
	AK172204	Zfp791
	AK172459	
	AK172630	
	AK181808	

	AK191498	
	AK194371	
	AK197973	
	AK198029	
	AK211473	
	AK216694	
	AW011738	
	AW112010	
	Acacb	
	Acsf2	
	Adamts13	
	Adgrf3	
	Adgrf4	
	Adhfe1	
	Afap1l2	
	Afmid	
	Aif1	
	Ak5	
	Akap3	
	Akt3	
	Alx3	
	Ankmy1	
	Ankrd42	
	Ano7	
	Ap1m2	
	Apod	
	Apol10b	
	Apol11b	
	Apol8	
	Apol9a	
	Apol9b	
	Apon	
	Aqp5	
	Arhgap27os3	
	Arhgef15	
	Art3	
	Asah2	
	Ascl3	
	Asgr2	
	Atp6v1g2	
	Atxn7l1os2	

	Axl	
	BB014433	
	BC030499	
	BC108341	
	Bcl2a1b	
	Bcl2a1d	
	Bco2	
	Best1	
	Btnl2	
	Btnl4	
	Btnl6	
	C130060C02Rik	
	C1qa	
	C1qb	
	C1qc	
	C1qtnf1	
	C1qtnf3	
	C1rl	
	C1s1	
	C1s2	
	C2	
	C230035I16Rik	
	C230037L18Rik	
	C8g	
	Cacng6	
	Camk2b	
	Car4	
	Casq1	
	Catsperg1	
	Ccdc183	
	Ccdc36	
	Ccl5	
	Ccm2l	
	Ccno	
	Ccr1	
	Ccr3	
	Ccr5	
	Ccrl2	
	Cd177	
	Cd300lf	
	Cd69	

	Cdkl3	
	Cep83os	
	Chac1	
	Chst1	
	Ciita	
	Clec11a	
	Clec12a	
	Clec1a	
	Clec4e	
	Clec4n	
	Clec7a	
	Clec9a	
	Clhc1	
	Clmn	
	Cmklr1	
	Cmpk2	
	Cnn3	
	Col1a1	
	Col2a1	
	Col6a1	
	Cpn2	
	Crp	
	Csf3r	
	Csmd2	
	Cybb	
	Cyp2c69	
	Cyp2d22	
	Cysltr2	
	D930028M14Rik	
	DQ551285	
	DQ715729	
	DQ717888	
	Dclk3	
	Dcst1	
	Ddx4	
	Ddx60	
	Dgka	
	Dgki	
	Dkk3	
	Dnah1	
	Dpf3	

	Dqx1	
	E030018B13Rik	
	E130114P18Rik	
	E230025N22Rik	
	E230029C05Rik	
	EG545963	
	Ear6	
	Edem2	
	Efemp2	
	Egr2	
	Eml2	
	Endou	
	Entpd1	
	Entpd3	
	Epha1	
	Espn	
	Etv4	
	Evl	
	Extl1	
	Fam149a	
	Fam178b	
	Fam187b	
	Fam19a2	
	Fam71f1	
	Fam78b	
	Fat3	
	Fbxo4	
	Fcgr1	
	Fgl2	
	Fgr	
	Flt4	
	Fndc5	
	Foxj1	
	Foxo6	
	Fut1	
	Fuz	
	Gal3st2	
	Gbp10	
	Gbp11	
	Gbp2b	
	Gbp7	

	Gbx1	
	Gfi1b	
	Gimap1	
	Gimap4	
	Gimap7	
	Gimap8	
	Gm10505	
	Gm10804	
	Gm11127	
	Gm12596	
	Gm13275	
	Gm13375	
	Gm14164	
	Gm15663	
	Gm16596	
	Gm16675	
	Gm17455	
	Gm17762	
	Gm19705	
	Gm2061	
	Gm2848	
	Gm4925	
	Gm5820	
	Gm614	
	Gm6432	
	Gm7244	
	Gm9961	
	Gng4	
	Gpha2	
	Gpr137c	
	Gpr161	
	Gpr174	
	Gpr31b	
	Gpx3	
	Gpx8	
	Grid1	
	Gucy2e	
	H2-Aa	
	H2-Ab1	
	H2-DMa	
	H2-DMb1	

	H2-DMb2	
	H2-Eb1	
	H2-Ob	
	H2-T24	
	Haao	
	Hcar2	
	Hck	
	Hcrt	
	Hdc	
	Heatr9	
	Hepacam	
	Hist1h2bk	
	Hmcn2	
	Hmgb4	
	Hoxb3	
	Hp	
	Hsd17b2	
	Hspa12a	
	Hspb1	
	Ido2	
	Ifi205	
	Ifi27l2a	
	Ifi44	
	Ifih1	
	Ifit1	
	Ifit2	
	Ifit3	
	Ifitm1	
	Igf2bp2	
	Igsf9	
	Il17d	
	Il27	
	Il2rb	
	Il33	
	Il9r	
	Inca1	
	Iqck	
	Irg1	
	Irgc1	
	Isg20	
	Islr	

	Itpka	
	Jsrp1	
	Kcnj6	
	Kcnk1	
	Kctd19	
	Klhl10	
	Klhl6	
	Klra2	
	Klrk1	
	Krt18	
	Lbp	
	Lhx2	
	Lif	
	Lincenc1	
	Lmntd2	
	Loxl2	
	Lrcol1	
	Lrfn1	
	Lrrc2	
	Lrrc48	
	Lrrc66	
	Lrrc73	
	Ltb	
	Ltb4r1	
	Ltbp1	
	Ltk	
	Ly86	
	Lyzl4	
	Mag	
	Marco	
	Mast4	
	Mcidas	
	Mdh1b	
	Mef2b	
	Mgat4e	
	Mgp	
	Mif1	
	Misp	
	Ms4a6c	
	Mttp	
	Muc20	

	Mx1	
	Myh11	
	Myom2	
	Mzf1	
	Naglu	
	Ncald	
	Nfkbie	
	Ninj2	
	Nipal1	
	Nod2	
	Notum	
	Nova2	
	Nr4a1	
	Nr4a2	
	Nr4a3	
	Nrg2	
	Nrn1	
	Nrp1	
	Ntng2	
	Oas1d	
	Oas1g	
	Oasl2	
	Olfr1444	
	Otc	
	Otop2	
	Otub2	
	Ovol1	
	P2rx3	
	Pax4	
	Pax8	
	Pcp4	
	Pcp4l1	
	Pde10a	
	Pde4b	
	Pde7b	
	Pecam1	
	Perp	
	Pgm5	
	Phf11a	
	Pipox	
	Plac8	

	Plag1	
	Plagl1	
	Plau	
	Plaur	
	Plbd1	
	Plekhg2	
	Plxnb3	
	Ppapdc2	
	Ppp1r3f	
	Ppp2r2b	
	Prg4	
	Prm1	
	Prob1	
	Prss36	
	Prtn3	
	Psd2	
	Ptges	
	Ptgis	
	Ptgs2	
	Ptgs2os	
	Ptgs2os2	
	Ptk6	
	Pvrl4	
	Pxylp1	
	Pycard	
	Pydc4	
	Rap1gap2	
	Rapgef3	
	Rasgef1b	
	Rfx8	
	Rgcc	
	Rgs9bp	
	Rilpl1	
	Ripply3	
	Rmi2	
	Rnf112	
	Rnf157	
	Rnf208	
	Rpp25	
	Rsad2	
	Rtp4	

	Rwdd2a	
	Rwdd3	
	Saa1	
	Scnn1a	
	Sectm1a	
	Sell	
	Selp	
	Sept3	
	Serpina11	
	Serpina3g	
	Serpina3h	
	Serpina3i	
	Serpina9	
	Serpind1	
	Serping1	
	Slamf6	
	Slamf7	
	Slc1a1	
	Slc22a21	
	Slc27a5	
	Slc28a3	
	Slc2a10	
	Slc2a6	
	Slc2a7	
	Slc30a2	
	Slc36a3	
	Slc3a1	
	Slc40a1	
	Slc6a19	
	Slc9a2	
	Slco2a1	
	Smco4	
	Sned1	
	Socs1	
	Sp6	
	Spata33	
	Spry4	
	Srms	
	Srpk3	
	St14	
	St3gal6	

	Styxl1	
	Sugct	
	Sulf2	
	Syngr3	
	Syt17	
	Tagap	
	Tbxas1	
	Tcp10b	
	Teddm1b	
	Tex22	
	Tgfb3	
	Thbs1	
	Timd4	
	Tlr12	
	Tmc3	
	Tmem102	
	Tmem211	
	Tmem220	
	Tmem231	
	Tmem37	
	Tmem53	
	Tmem86b	
	Tmem8b	
	Tmprss6	
	Tmsb10	
	Tnfaip2	
	Tnfaip3	
	Tnfrsf14	
	Tnfsf10	
	Trap1a	
	Trim34a	
	Trim72	
	Trp53cor1	
	Trpc2	
	Trpc5os	
	Trpm6	
	Tspyl4	
	Tuba3a	
	U66835	
	Upp1	
	Ushbp1	

	Vasn	
	Vdr	
	Vegfa	
	Vmn2r-ps129	
	Vps37d	
	Wfdc17	
	Xkrx	
	Ypel4	
	Zbp1	
	Zbtb3	
	Zc3h6	
	Zfp109	
	Zfp133-ps	
	Zfp174	
	Zfp389	
	Zfp532	
	Zfp819	
	Zfr2	
	Zmat1	

**Supplementary Table 4. Gene Subset Involved in at least one Pro-Inflammatory Biological Process.** These 99 genes are present in at least one of the 5 enriched biological processes mentioned in Table 2.
**Gene Name**
2010002M12Rik
Afap1l2
Aif1
Axl
Bcl2a1d
C1qa
C1qb
C1qc
C1rl
C2
C8g
Ccl5
Ccr1
Ccr3
Ccr5
Ccrl2
Cd300lf
Chst1
Ciita
Clec4e
Clec4n
Crp
Csf3r
Cybb
Ddx60
Endou
Fcgr1
Fgr
Foxj1
Gbp7
Gfi1b
Gimap1
Gm11127

Gm13275
Gpr174
H2-Aa
H2-Ab1
H2-DMa
H2-DMb1
H2-DMb2
H2-Eb1
H2-T24
Hcar2
Hck
Heatr9
Hist1h2bk
Hoxb3
Hp
Ido2
Ifih1
Ifit1
Ifit2
Ifit3
Ifitm1
Il27
Il2rb
Il33
Irg1
Isg20
Klhl6
Klrk1
Lbp
Lif
Ltb
Ly86
Marco
Mx1
Nod2
Nr4a3
Oasl2
Pde4b
Pecam1

Plac8
Prg4
Prtn3
Ptgs2
Ptk6
Pycard
Rgcc
Rsad2
Saa1
Sectm1a
Sell
Selp
Serpina3g
Serping1
Slamf6
Slamf7
Slc40a1
Srms
Tagap
Thbs1
Tlr12
Tnfaip3
Tnfrsf14
Tnfsf10
Trim34a
Vegfa
Zbp1

**Supplementary Table 5. List of 689 Genes that are down-regulation by IFN and not reversed by ONX**
**Gene Name**
1500017E21Rik

1600010M07Rik
1600014K23Rik
1700012B07Rik
1700012L04Rik
1700067K01Rik
1810011H11Rik
1810011O10Rik
1810022K09Rik
1810024B03Rik
2010107E04Rik
2010204K13Rik
2210039B01Rik
2310002L09Rik
2310015A10Rik
2310045N01Rik
2810417H13Rik
2810442N19Rik
3110045C21Rik
3300005D01Rik
4632428N05Rik
4732416N19Rik
4921531C22Rik
4930447F24Rik
4930513N10Rik
4930525D18Rik
4930577N17Rik
5430425K12Rik
5830432E09Rik
6230400D17Rik
8030423J24Rik
8430408G22Rik
9530027J09Rik
A130077B15Rik
A430078G23Rik
A630066F11Rik
A930018M24Rik
AI661453
AK006651
AK008665
AK008812
AK013883

AK015284
AK018753
AK030770
AK031066
AK034234
AK034420
AK035263
AK039009
AK039019
AK039370
AK041539
AK041758
AK044938
AK045164
AK046742
AK053029
AK054476
AK077171
AK078239
AK078466
AK078801
AK079017
AK083675
AK084265
AK084393
AK085274
AK089049
AK131873
AK135766
AK138590
AK141314
AK144545
AK144695
AK147021
AK154077
AK154309
AK160668
AK162138
AK162791
AK162806
AK164636

AK165532
AK170266
AK171855
AK171960
AK186650
AK189470
AK202078
AK202494
AK209647
AK209885
AK217047
Aacs
Abca6
Abca9
Abcd2
Acadm
Acat2
Accsl
Acsl3
Actb
Actg2
Actn1
Actn2
Adam8
Adat3
Adcy9
Adgre5
Adk
Adrb2
Agmo
Agt
Aire
Ak4
Akap13
Akap8
Aldh3a1
Aldh4a1
Ambp
Amd1
Amhr2
Ang

Ang2
Angpt2
Ankrd37
Anxa1
Anxa3
Apex1
Apln
Apoc1
Apoc2
Apoc4
Aqp1
Aqp8
Arg1
Arglu1
Arhgap19
Arhgap31
Arl15
Asb10
Asb2
Asb4
Asb5
Ascl1
Atg9b
Atp5g1
Atp5j2
Atp5k
Atp7b
BB123696
BC021767
BC029853
BC031361
BC031727
BC039771
BC048080
BC048859
BC100451
Bcam
Bco1
Bmper
Bnip3
Bop1

Brwd1
Btbd17
C5ar2
Calm2
Caln1
Calr4
Camk1
Casp6
Cbr2
Ccdc113
Ccdc116
Ccdc129
Ccdc134
Ccdc167
Ccl9
Ccnf
Cd101
Cd109
Cd163
Cd2
Cd207
Cd28
Cd300lb
Cd300ld
Cd5l
Cd84
Cd9
Cdc20
Cdc25c
Cdhr2
Cdk5r1
Cdkn3
Celf2
Cfap57
Cgnl1
Chst13
Cisd3
Cks2
Clcn3
Clcn5
Cldn11

Clec4a1
Clec4a2
Clec4a3
Clint1
Cndp1
Cnr2
Col12a1
Col4a3bp
Col6a5
Coro2b
Cotl1
Cox6b1
Cox7a1
Cox7a2
Cox7b
Cpeb2
Cpne5
Cpne9
Cpxm1
Csf1r
Ctsk
Cxcr2
Cyp4f18
Cyp4f37
Cyp51
Cysltr1
Cytip
Dab2
Dad1
Daglb
Dbi
Dclk1
Ddc
Ddx39
Ddx3x
Ddx46
Dennd4c
Depdc1a
Dhrs3
Dhrs9
Dhx15

Dhx9
Dlg3
Dnah6
Dnajc25
Dstn
Dusp27
Dusp4
Egln1
Egln3
Elane
Ell2
Elovl6
Emid1
Emp1
Enpep
Epas1
Epm2a
Erg
Erv3
Ets1
Etv5
F10
F13a1
F2rl3
F9
FJ040209
FJ422280
Fabp4
Fads6
Fam133b
Fam170b
Fam198b
Fam20a
Fam20c
Fam221b
Fam222a
Fam43a
Fam50b
Fam83d
Fam84b
Fasn

Fbln1
Fcnb
Fcrls
Fdft1
Fdps
Fgd4
Fgd5
Flt1
Fmo9
Fmr1
Folr1
Frmd6
Frmpd1
Fubp1
G2e3
Gal3st1
Galr3
Gcat
Gfra1
Gldn
Gli1
Gm11710
Gm13889
Gm14327
Gm14461
Gm16062
Gm1653
Gm4890
Gm572
Gm6093
Gm6116
Gm6682
Gm684
Gm7854
Gmpr
Gpd1
Gpr155
Gpr183
Gpr20
Gpr62
Gprc5c

Grhl3
Grhpr
Grin1
Gsg1
Gsn
Gstm2
Guca1b
Guca2b
H1f0
H1foo
H2-M5
H2afy3
Havcr2
Hdgfrp3
Hfe
Hist1h1c
Hist1h3g
Hist1h4a
Hist2h3c1
Hivep3
Hmgb3
Hmgcr
Hmgcs1
Hnrnpd
Hnrnph1
Hnrnpll
Hnrnpm
Hopx
Hpgds
Hsd17b7
Icam2
Icam4
Idh1
Idi1
Ids
Ier3
Ifi27
Ifitm10
Ifngr1
Igf1
Igfbp4

Igsf6
Il1b
Il1r1
Il1rap
Il1rl1
Il20rb
Insig1
Ipcef1
Iqcf1
Iqsec3
Ivl
Jph3
Kbtbd8
Kcne3
Kcnj12
Kcnk6
Kif20a
Kif21a
Kif4
Klf4
Klhl30
Klhl33
Klk1b11
Kmt2e
Kpna2
Krt7
Kti12
Lama4
Lamb1
Lbr
Lcp1
Ldlrad3
Lilrb4
Lingo1
Lipn
Lmcd1
Lpar5
Lpcat1
Lpxn
Lrrc23
Lsp1

Lss
Ltc4s
Ly96
Lyz1
Lyz2
Mafb
Magoh
Mamdc2
Man1a
Mapt
Mat2a
Matk
Matn1
Mcemp1
Mei1
Metrnl
Mfsd6l
Mfsd7c
Mgl2
Mgll
Mki67
Mkl2
Mmp8
Mmp9
Mns1
Mog
Mpzl2
Mrc1
Mroh2a
Mrpl18
Mrpl34
Mrps21
Mrvi1
Ms4a7
Msmo1
Mvd
Mxd4
Mylip
Myo15b
Myom1
Myom3

Naa50
Ncbp2
Ndst3
Ndufa4
Ndufb3
Nek3
Nfam1
Nkain1
Nlrp10
Nme1
Nme3
Nop10
Nop58
Notch4
Noxo1
Nradd
Nsdhl
Ntf3
Nuak1
Nxt2
Oit3
Olfr110
Olfr111
Olfr112
Olfr114
Ost4
Otoa
Otud6a
P2ry1
P2ry13
P2ry6
P4htm
Padi1
Pald1
Papln
Pax3
Pbk
Pbp2
Pcyt2
Pdp2
Pdzk1

Pet100
Pgp
Phlda2
Pi16
Pif1
Pik3c2g
Pik3cg
Plekha3
Plk1
Polr2d
Polr2l
Polr3g
Pou2af1
Pparg
Ppp1r37
Pradc1
Prc1
Prg2
Proz
Prpf4b
Ptch2
Ptchd1
Ptgds
Ptgs1
Purb
Rab44
Rangrf
Rasgrp3
Rbm12
Rdh11
Rep15
Rex2
Rgs11
Rgs18
Rgs8
Rhoj
Rhox5
Rimkla
Rnase2a
Rnase4
Rnaseh2c

Rnf145
Romo1
Rpgrip1
Rph3al
Rtn4rl1
S100a8
S1pr1
Samsn1
Sardh
Sbk1
Sbsn
Sc5d
Scara5
Scd2
Scd3
Scd4
Scn3a
Sdc4
Sdhd
Sec14l2
Sec61b
Selenbp2
Sema4g
Sema5b
Sephs2
Serpinf1
Sfpq
Sgsm1
Sigmar1
Skp1a
Skp2
Sla
Slc12a2
Slc13a3
Slc16a8
Slc17a5
Slc24a5
Slc25a10
Slc26a11
Slc2a9
Slc36a2

Slc39a10
Slc47a1
Slc4a2
Slc6a12
Slc6a13
Slc6a6
Slco4a1
Smap2
Smim11
Smoc2
Snai2
Snrnp25
Snrpd1
Snrpd3
Spink5
Sptssa
Sqle
Srsf1
Srsf2
Srsf3
Srsf6
Stard4
Syn2
Tbc1d16
Tcp11l2
Tcstv1
Tecta
Tfec
Tg
Tlr13
Tlr7
Tlr8
Tm4sf19
Tmcc2
Tmem26
Tmem97
Tnnc2
Tnni2
Toporsos
Tpcn1
Trabd2b

Trem1
Trem3
Trim59
Trim66
Tspan10
Tuba1c
Tuba8
Txndc11
Ubald1
Ube2n
Ucn2
Umodl1
Uqcrb
Uqcrh
Usmg5
Utp14b
Vat1l
Vwa5b1
Wdfy2
Wdfy4
Wdr26
Wdr6
Wdr83os
Wee1
Wnt5b
Wsb2
Xk
Xpnpep1
Xrcc5
Ydjc
Zbtb8os
Zfp207
Zfp558
Zfp575
Zfp609
Zfp672
Zfp931
Znrf2
